# Hybridization and diversity of the genus *Vandenboschia* in Korea insights from morphological, cytological, and genotype analyses

**DOI:** 10.1038/s41598-025-86000-3

**Published:** 2025-01-10

**Authors:** Sang Hee Park, Jung Sung Kim, Hyoung Tae Kim

**Affiliations:** 1 Department of Forest Science, Chungbuk National University, Cheongju, 28644 Chungbuk Korea; 2 Department of Crop Science, Kyungpook National University, Sangju, 37224 Kyungpook South Korea

**Keywords:** *Vandenboschia radicans* complex, CAPS, Genotype, Morphology, Hybrid, Ploidy, Plant sciences, Plant evolution, Phylogenetics, Speciation, Taxonomy

## Abstract

The genus *Vandenboschia* exhibits significant diversity, driven by complex hybridization events and varying ploidy levels in the natural habitats of Korea, leads to misidentifications that obscure its true distribution and classification. To address this issue, the present study employed morphological, cytological, and genotype analyses to clarify the taxonomical circumscription and distribution patterns of the *Vandenboschia radicans* complex in Korea. The *V. radicans* complex in the genus refers to *V. radicans* and its closely related species and exhibits significant morphological variation, making classification challenging. Previous molecular studies have revealed that it consists of several non-hybrid and hybrid species among them. Through the present study, comprehensive sampling from Jeju and Ulleung Islands identified five distinct taxa from the *V. radicans* complex: two non-hybrid species (*Vandenboschia kalamocarpa* and *Vandenboschia nipponica*), and three hybrids (*Vandenboschia × stenosiphon*, *Vandenboschia × quelpaertensis*, and *Vandenboschia kalamocarpa* × *Vandenboschia nipponica* × *Vandenboschia striata*). Notably, hybrids exhibited broader geographic distributions compared to non-hybrid species, which were confined to specific microhabitats. While overlapping morphological traits among the species complicate identification without genetic analysis, rhizome diameter and the width-to-length ratio of the involucre can serve as key morphological traits for distinguishing members of the *Vandenboschia radicans* complex in Korea. Additionally, cleaved amplified polymorphic sequence (CAPS) markers and cloning techniques were employed for species identification, particularly when morphological traits were inconclusive. These findings highlight the need to integrate genetic tools with traditional taxonomic methods to resolve complex species relationships within *Vandenboschia* in Korea and emphasize the importance of conservation efforts for geographically restricted and genetically distinct populations.

**Supplementary Information:**

The online version contains supplementary material available at 10.1038/s41598-025-86000-3.

## Introduction

The family Hymenophyllaceae Mart., a primary group within the basal leptosporangiate ferns, comprises approximately 600 species across nine genera, distributed in both tropical and temperate regions worldwide^1,2^. Members of this family are generally epiphytic and are characterized by leaf blades typically composed of a single cell layer, with sporangia forming in receptacles located within the involucres at the segment margins^1,2^. Their spores are chlorophyllous^2,3^, and many taxa are known to have long-lived gametophytes in the form of ribbons or filaments^4–6^.

*Vandenboschia* Copel., one of the nine genera within the family Hymenophyllaceae, is distributed across tropical, subtropical, and northern temperate regions^1^. This genus is primarily hemi-epiphytic or epilithic, characterized by numerous roots, a creeping rhizome, and a tubular to cup-shaped involucre with a long projecting receptacle^1,7^.

The classification of Hymenophyllaceae has been approached from various perspectives due to the difficulty in morphological classification. Traditionally, Hymenophyllaceae has been divided into two genera: *Hymenophyllum*, with bivalved involucres, and *Trichomanes*, with tubular ones^1,8^. Copeland^8^ reclassified Hymenophyllaceae into 33 genera, introducing *Vandenboschia* Copel., characterized by its epiphytic nature, elongated rhizomes, remote and pinnately dissected fronds, uniformly thin cell walls, funnel-shaped involucres, and protruding receptacles with small sporangia. He noted that *Vandenboschia* is closely related to the primitive form of the *Trichomanes* clade, which includes many cosmopolitan species, and designated *Vandenboschia radicans* (Sw.) Copel. from the West Indies as the type species of the genus.

Subsequently, Morton^9^ organized Hymenophyllaceae into six genera, reclassifying most of Copeland’s *Vandenboschia* into *Trichomanes* sec. *Lacosteopsis*, featuring epiphytic plants with creeping rhizomes, bi- to quadri-pinnatifid, non-ciliated fronds, and leaf blades lacking false veins, with uniformly shaped leaf cells. However, these features were not significantly different from those of Copeland’s *Vandenboschia*. Following this, Iwatsuki^10,11^ classified the family into eight genera, moving *Vandenboschia* species into *Crepidomanes* sec. *Maiora*.

More recently, Hymenophyllaceae was reclassified into nine genera based on molecular data analysis, leading to the reintroduction of the genus *Vandenboschia*. This reclassification was necessary because the species of Copeland’s *Vandenboschia* were polyphyletic, resulting in the reassignment of all species, except for *V. radicans* and its relatives, to other genera^1^. However, identifying species within *Vandenboschia* remains challenging due to wide morphological variation within a species and overlapping morphological characteristics among species. Consequently, *V. radicans* and its close relatives, which are widely distributed in tropical and northern temperate regions, are referred to as the “*Vandenboschia radicans* complex”^12^.

Ito^13^ categorized the *V. radicans* complex distributed in Japan into three varieties based on leaf size: large, medium, and small types. Since then, these three taxa have been maintained with changes in their scientific names, including the very small species, *Vandenboschia subclathrata*, found in the Yaeyama Islands^14,15^. However, observations of numerous specimens displaying intermediate characteristics between these types^12^ and frequent reports of sterile triploids exhibiting irregular meiotic behavior in Japanese taxa^16,17^ have raised the possibility of hybridization among them^18^.

To enhance species discrimination of the *V. radicans* complex in Japan and adjacent areas, comprehensive analyses were undertaken, including ploidy level confirmation and genotyping using *GapCp*, a single-copy nuclear DNA region, and the chloroplast *rbcL* gene^12^. These analyses identified diploid, triploid, and tetraploid individuals within the complex, with triploids being the most dominant and diploids being relatively rare^12^. Overall, except for *Vandenboschia liukiuensis* (Y.Yabe) Tagawa, which is clearly distinguishable by its involucre characteristics, the diversity of the *V. radicans* complex in Japan and adjacent areas was interpreted as a result of reticulate evolution involving hybridization and polyploidization among three diploid species^12,19^. Combining these genetic data with morphological traits, four non-hybrid diploid species were identified: *Vandenboschia kalamocarpa* (Hayata) Ebihara with medium-sized leaves (αα genome), *Vandenboschia nipponica* (Nakai) Ebihara with small-sized leaves (ββ genome), *Vandenboschia striata* (D.Don) Ebihara with large-sized leaves (γγ genome), and *Vandenboschia subclathrata* K. Iwats. with small-sized leaves (αα genome) in the Yaeyama Islands^19^. Additionally, four sterile hybrids and four allotetraploid species generated by hybridizations between diploid species were also recognized^19^.

Through the aforementioned studies, it was concluded that the *V. radicans* complex in Japan primarily consists of a triploid species, while the original diploid species have a more restricted distribution^19,20^. The findings of these studies emphasize the importance of confirming genotype and ploidy level for accurate identification, due to the extensive morphological variation among hybrid species^12,19,20^.

In the case of Korean taxa, three species—*Trichomanes amabile* Nakai, *Trichomanes quelpaertense* Nakai, and *Trichomanes stenosiphon* Christ—were described in the early 1900s, with Jeju Island as the type locality (Supplementary Table 1). These three species were initially reported by Nakai^21^ in his survey of Korean flora; however, only *Trichomanes orientalis* C. Chr was subsequently listed by Chung^22^ in his floral survey. Subsequently, Park^23^ later distinguished two species, the “medium-sized with flat leaves” *Vandenboschia radicans* var. *orientalis* (C. Chr.) H. Ito, and the “small-sized with three-dimensional leaves” *V. amabilis* (Nakai) K. Iwats., aligning with Ito^13^’s classification based on leaf size (e.g., “small,” “medium,” and “large”). Sun^24^ and Moon^25^ maintained these classifications of “medium” and “small” species proposed by Park^23^, but updated their scientific names to *Crepidomanes radicans* (Sw.) K. Iwats. and *Crepidomanes amabile* (Nakai) K. Iwats., respectively, following Iwatsuki^10^’s guidelines. Park, et al.^26^ also recognized these two species, designating them as *Lacosteopsis orientalis* (C. Chr.) Nakaike and *Lacosteopsis orientalis* var. *abbreviata* (C. Chr.) Nakaike, based on Nakaike^15^’s interpretations.

Interestingly, despite Jeju Island being designated as its type locality^21^, the recognition of *T. quelpaertense* in Korea had disappeared, except for its type collection^19^. More recently, Lee and Lee^27^ adopted Ebihara, et al.^19^’s taxonomical framework, assigning *Vandenboschia kalamocarpa* (Hayata) Ebihara to the “medium” species and *Vandenboschia nipponica* (Nakai) Ebihara to the “small” species. They also recognized two hybrids, *Vandenboschia* × *stenosiphon* (Christ) Copel. and *Vandenboschia* × *quelpaertensis* (Nakai) Ebihara, both of which had Jeju Island as their type locality. Given the morphological similarities among these taxa, confirming the genotype and ploidy is crucial for accurate species identification within the genus *Vandenboschia*, as emphasized in previous studies by Ebihara et al.^12,19^. However, in Korea, classification within this genus has primarily adhered to the morphological distinction of “medium” and “small” species based on leaf sizes, without genotype or ploidy confirmation, relying solely on the scientific names proposed by Ebihara, et al.^19^.

Although the scientific names of the two original Korean taxa have been updated to *V. kalamocarpa* and *V. nipponica*, there remains a significant risk of misidentifying *Vandenboschia* species without additional information. Recent studies by Japanese researchers suggest a different scenario: only two hybrid species, *V*. × *stenosiphon* and *V*. × *quelpaertensis*, are found in Korea, excluding *V*. *kalamocarpa* and *V. nipponica.* Specifically, *V. nipponica* is recognized as an endemic fern to Japan^12,19,28^. This discrepancy in the identification of the *Vandenboschia radicans* complex between adjacent countries may stem from the lack of cytological and molecular data on Korean *Vandenboschia* species.

As morphological identification of the *V. radicans* complex remains contentious^12,19,29^, molecular identification becomes crucial to accurately determining species using single nucleotide polymorphisms (SNPs). Unfortunately, the reticulate evolution of this complex hinders the direct sequencing of general PCR products for a single copy nuclear gene due to the heterogeneity of alleles. Therefore, single-strand conformation polymorphism (SSCP) has been proposed as a suitable method to confirm genotypes^12^. SSCP can detect SNPs and small mutations across broad regions by focusing on the conformational changes in single-stranded DNA. However, this technique requires careful optimization of electrophoresis conditions. Conversely, cleaved amplified polymorphic sequence (CAPS) is a simple and cost-effective method for detecting known polymorphisms that affect restriction sites. This codominant marker is widely used for identifying genotypes of various plant species^30–34^.

This study aimed to achieve the following objectives through extensive sampling of the *V. radicans* complex in Korea: (1) Introduce CAPS markers capable of rapidly identifying the genotypes proposed by Ebihara, et al.^12^, and assess their effectiveness and accuracy; (2) Definitively confirm the *Vandenboschia* species distributed in Korea through genotyping, measuring morphological characters, and determining ploidy levels; and (3) Discuss the characteristics and distribution patterns of each identified species in Korea.

## Materials and methods

### Extensive sampling of *V. radicans* complex

**Fig. 1:**
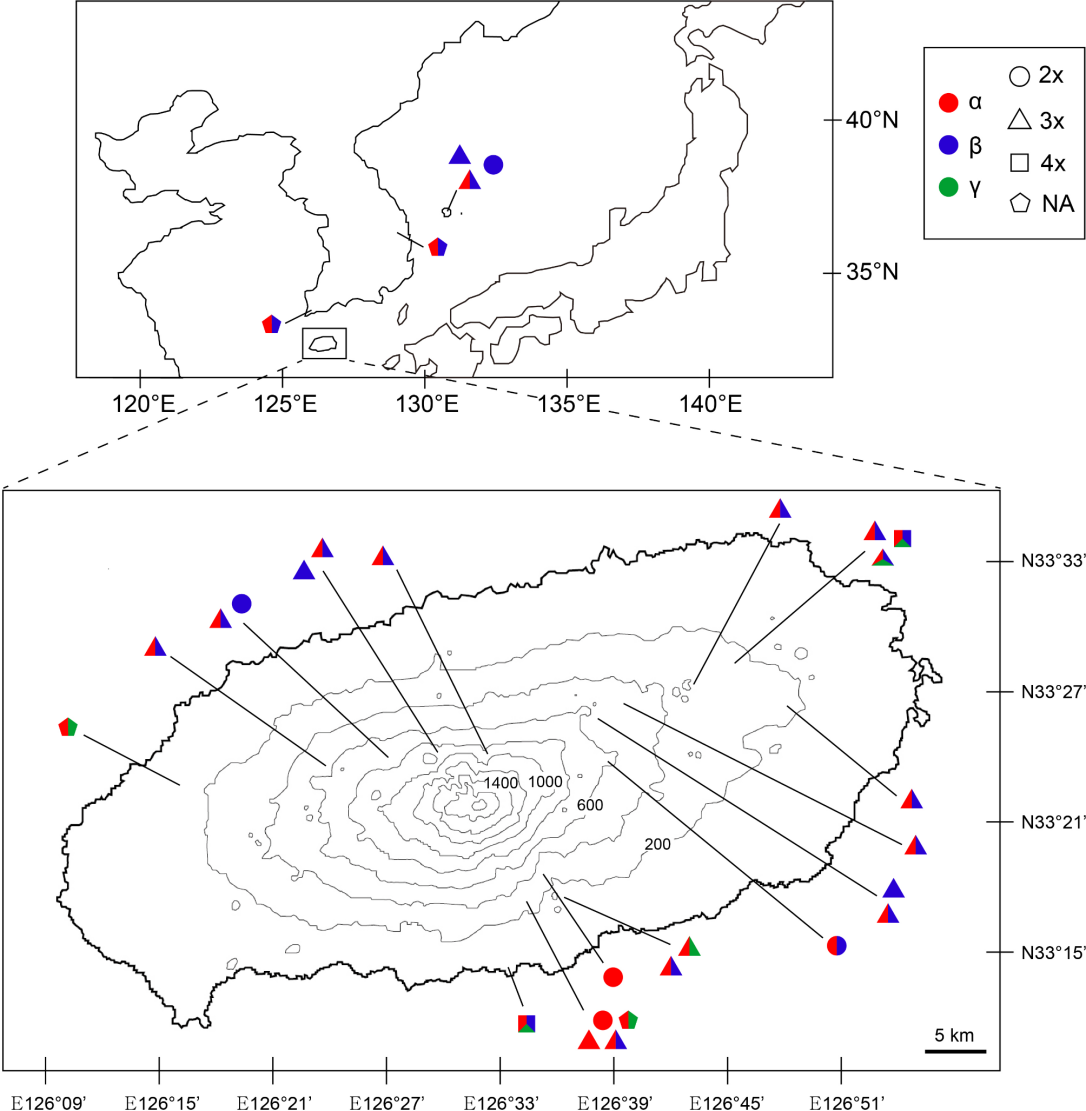
Distribution of the sample collecting sites and the genotype compositions of the species analyzed in the present study.

A total of 63 individuals of *Vandenboschia* were used as plant materials in this study. Of these, 59 samples were collected from their natural habitats on Jeju Island, located southwest of the Korean Peninsula, and Ulleung Island in the East Sea (Fig. 1). The remaining four samples were obtained from herbarium specimens preserved at Korean herbaria, the Korea National Arboretum (KNA) and National Institute of Biological Resources (NIBR). We also attempted multiple field investigations on and around the recorded collection sites, as described in the labels of four specimens analyzed in the present study, to collect living samples from inland areas of the Korean Peninsula to search for new habitats and re-explore these areas (Fig. 1). However, it was impossible to find additional populations of *Vandenboschia* species at those sites.

Due to their long creeping rhizome and epilithic growth form, accurate field identification was challenging. Therefore, a mass of rhizomes with fronds from a single rock slope was sampled and stored in separate bottles in a plant incubator for subsequent experiments. Voucher specimens consisting of rhizomes with fronds were selected from the bottles and deposited in the herbarium of Chungbuk National University (CBNU) under its own specimen number (Supplementary Table 2). There was no permission required to collect the plant materials for this study and they were primarily identified in the field based on the morphological characteristics by S. H. Park until final confirmation. All procedure for the study including colleting materials, making voucher specimen, and managing the living materials were performed in accordance with the guideline of Chungbuk National University.

### Genome size measurement and chromosome counting of *Vandenboschia*

A fresh leaf from each individual was finely chopped along with a fresh leaf of *Nicotiana tabacum* L. (1 C = 4.6 pg, Zonneveld, et al.^35^) in MB01 nuclear extraction buffer^36^. The extracted nuclei were stained with a UV precise P staining buffer containing DAPI (4′,6-diamidino-2-phenylindole, Sysmex-Partec, Munster, Germany), and subsequently analyzed using a CyFlow^®^ Ploidy Analyzer (Sysmex-Partec, Munster, Germany). Genome sizes were calculated relative to the 1 C-value of *N. tabacum*.

To confirm the chromosome number, healthy root tips were pretreated with a 2 mM 8-hydroxyquinoline solution overnight at 23 °C and then fixed in a solution of ethanol and acetic acid (3:1, v/v) for 4 h at 4 °C. After fixation, the root tips were transferred to 70% ethanol and stored at -20 °C until further processing. The fixed root tips were macerated in 1 N HCl for 3 min at 60 °C, squashed, and finally stained using 2% aceto-orcein. The mitotic metaphase chromosomes were observed at 1,000× magnification using a light microscope (Olympus BX50, Tokyo, Japan).

### DNA extraction and PCR amplification for genotyping

DNA was extracted from the same individual leaves used for genome size determination by flow cytometry using the DNeasy Plant Mini Kit (Qiagen, Hilden, Germany), according to the manufacturer’s protocol. The chloroplast *rbcL* region was amplified using the primer set *rbcL*-TKT-F1 and *rbcL*-TKT-R3N-2 ^12^. The reaction mixture comprised 10 µL of AccuPower^®^ PCR Premix (Bioneer, Daejeon, Korea), 1 µL of each primer (10 pM), 0.5 µL of DNA, and distilled water to bring the total volume to 20 µL. The PCR conditions were as follows: initial denaturation at 95℃ for 5 min, followed by 30 cycles of denaturation at 95℃ for 45 s, annealing at 57℃ for 20 s, extension at 72℃ for 60 s, and a final extension at 72℃ for 10 min. The amplified PCR products were purified using Expin™ PCR SV (GeneAll, Seoul, Korea), and then sequenced on the AB1 3730xl system at Macrogen, Seoul, Korea.

The nuclear *GapCp* region was amplified using the primer set *GapC*-7FA and *GapC-*BR1 ^12^. To minimize polymerase errors, Phusion™ Plus PCR Master Mix (ThermoFisher Scientific, Waltham, USA) was used for amplification. The reaction mixture comprised 10 µL Phusion mixture, 1 µL of each primer (10 pM), 1 µL of DNA, and distilled water to bring the total volume to 20 µL. The PCR conditions were as follows: initial denaturation at 98℃ for 5 min, followed by 30 cycles of denaturation at 98℃ for 30 s, annealing at 60℃ for 20 s, extension at 72℃ for 60 s, and a final extension at 72℃ for 10 min. The amplified PCR products were purified using PureLink™ PCR purification kit (Invitrogen, Waltham, USA).

For hybrid species, PCR products of the nuclear *GapCp* region were cloned to sequence each genotype individually. Cloning was performed using the Zero Blunt™ TOPO™ PCR Cloning Kit (Invitrogen, Waltham, USA), following the manufacturer’s protocol. At least 2.5 times the number of colonies corresponding to the ploidy level of each sample were selected for analysis. Each colony was purified using Expin™ PCR SV (GeneAll, Seoul, Korea) and sequenced using an *M13F* universal primer on an AB1 3730xl system at Macrogen, Seoul, Korea. When the number of identified genotypes matched the ploidy level of each sample, the precise genomic formula was determined (e.g., AAB or ABB). However, if the number of genotypes was confirmed to be less than the ploidy level, the genomic formula for that sample was indicated with an asterisk (e.g., A*B).

Combining these two approaches, the genotype of each was assigned using the symbols α, β, γ, and * to indicate unknown. The first character, underlined (_), represented the maternal genome type, refining the method of Ebihara, et al.^12^.

### Designing CAPS markers and conducting CAPS experiments

**Fig. 2:**
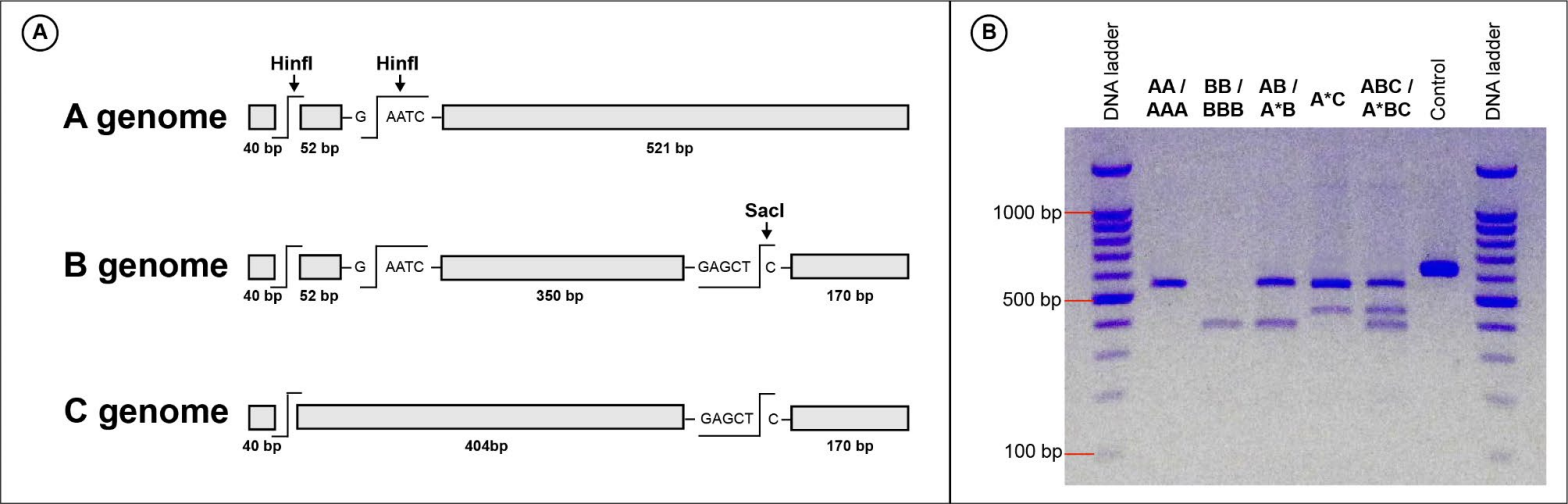
CAPS markers of the nuclear *GapCp* region distinguishing the α, β, and γ genomes using the restriction enzymes SacI and HinfI. (**A**) Schematic representation of genotype analysis using CAPS markers. **(B)** Electrophoretic patterns produced by CAPS for each genotype.

To design the CAPS markers, all *GapCp* sequences produced by Ebihara, et al.^12^ were downloaded from the NCBI (National Center for Biotechnology Information) GenBank and aligned using the alignment software, MUSCLE^37^. Potential restriction enzyme sites that could distinguish among A, B, and C genotypes of *GapCp* were identified using Geneious Prime^®^ version 2023.0.4 (Auckland, New Zealand). As a result, two restriction enzymes, SacI and HinfI, were selected for their ability to differentiate these genotypes. The expected digestion results for each genotype are shown in Fig. 2A. The amplification and purification of the *GapCp* region for CAPS analysis followed the same conditions as previously described. The purified PCR products were digested with 3U of SacI and HinfI, respectively. The resulting band patterns were examined through electrophoresis on a 2% agarose gel using 0.5X TBE buffer (Fig. 2B). To simplify the band patterns, short DNA fragments of 40 bp shared among the three genotypes and 52 bp fragments commonly found in A and B genotypes cut by HinfI were ignored.

### Measurement of morphological traits

**Fig. 3:**
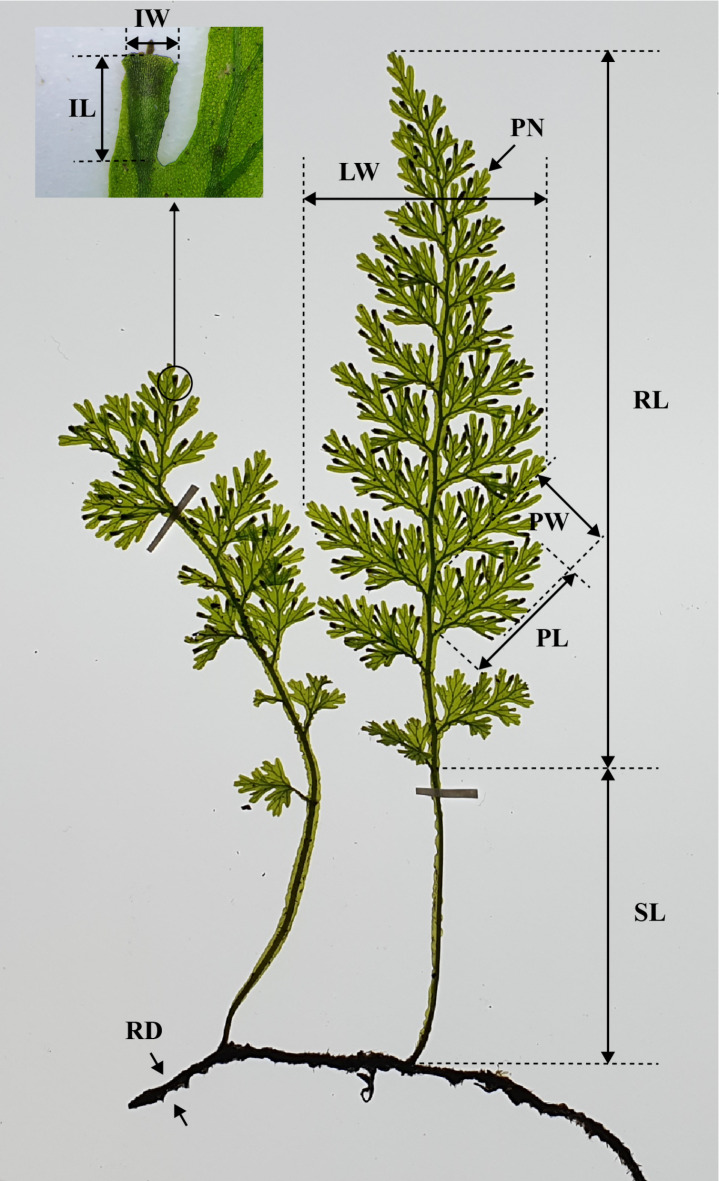
Morphological traits measured in this study and their abbreviations. RD: rhizome diameter; SL: stipe length, RL: rachis length; LW: maximum leaf blade width; PL: maximum pinna length; PW: maximum pinna width; PN: pinnae number; IL: involucre length; IW: involucre width.

Of the 63 individuals, 47 were used for measuring the morphological traits, as the ploidy information for the remaining 16 individuals was not available. To analyze the morphological variation within the genus, ten morphological traits were measured from individuals with mature leaves bearing involucres, and the averages of each trait were calculated (Fig. 3). These traits included rhizome diameter (RD), stipe length (SL), rachis length (RL), maximum leaf blade width (LW), maximum pinna length and width (PL, PW), pinnae number (PN), and involucre length and width (IL, IW). The RD was measured at random points along the creeping rhizome using a digimatic caliper (CD-15APX, Mitutoyo, Japan). The six leaf traits of leaf (SL, RL, LW, PL, PW, and PN) were assessed from mature leaves with involucres. Measurements of the involucre characteristics (IL and IW) were obtained by averaging the dimensions of up to 10 involucres per specimen.

Both of *α*αγ and *α*β genotypes were identified in only one individual, and developed involucres were ansent in the *α*β genotype. Therefore, the *α*β genotype was excluded from the measurement of morphological traits, and the *α*αγ genotype was used as a reference material for featuring the γ genotype. Finally, the 10 morphological traits were measured in four groups, excluding the *α*αγ and *α*β genotypes (Table 1): G-1 (*V. kalamocarpa*: *α*α/*α*αα), G-2 (*V. nipponica*: *β*β/*β*ββ), G-3 (*V. × stenosiphon*: *α*αβ, *α*ββ, *α*β*/βαα, *β*βα, *β*α*), and G-4 (*V. kalamocarpa* × *V. nipponica* × *V. striata*: *γ*βα/*α*αβγ/*α*ββγ).

**Table 1:** Morphological trait measurements of Korean *Vandenboschia* by genotypes.

Genotype	RD (mm)	SL (mm)	RL (mm)	LW (mm)	PL (mm)	PW (mm)	PN	IW (mm)	IL (mm)	IW / IL
I (αα/ααα)	0.43 ± 0.01	15.92 ± 3.47	32.29 ± 1.11	16.71 ± 1.25	10.04 ± 0.67	6.46 ± 0.84	7.12 ± 0.37	0.75 ± 0.05	1.42 ± 0.12	0.53 ± 0.01
II (ββ/βββ)	0.41 ± 0.02	16.68 ± 12.53	49.98 ± 22.26	17.89 ± 6.43	9.22 ± 3.57	6.49 ± 2.34	10.41 ± 1.97	0.84 ± 0.08	0.98 ± 0.09	0.85 ± 0.05
III (*α*β*^1)^/*β*α*^2)^)	0.42 ± 0.05	29.04 ± 16.63	75.01 ± 39.31	24.45 ± 7.59	14.50 ± 5.70	9.80 ± 2.98	10.86 ± 2.55	0.78 ± 0.10	1.46 ± 0.18	0.53 ± 0.06
*α*αγ^3)^	0.57	31.5	64.0	33.5	19.5	12.25	10.0	0.75	1.74	0.43
IV (*γ*αβ/*α*βγ*^4)^)	0.65 ± 0.02	54.03 ± 19.47	137.27 ± 27.97	48.77 ± 2.71	27.07 ± 2.14	15.80 ± 2.87	15.27 ± 1.91	0.70 ± 0.14	1.79 ± 0.32	0.39 ± 0.02

RD: rhizome diameter; SL: stipe length; RL: rachis length; LW: maximum leaf blade width; PL: maximum pinna length; PW: maximum pinna width; PN: pinna number; IW: involucre width; IL: involucre length.

(1) Include *α*αβ, *α*ββ, *α*β*.

(2) Include *β*αα, *β*βα, *β*α*.

(3) There is only one measurable sample available.

(4) Include *α*αβγ, *α*ββγ.

To compare the mean values of the four groups for each morphological trait, normality and homogeneity of variances were checked. Then, Normal-ANOVA and Tukey’s HSD were used for data with normal distribution and equal homogeneity of variances, while the Kruskal-Wallis test and Dunn’s test with Holm correction were used for data with non-normal distribution.

Based on the eight traits, excluding IL and IW but including the width-to-length ratio of the involucre, discriminant analysis of principal components (DAPC) was conducted to determine if the four genotypic groups could be distinguished from one another. The original variables were scaled prior to analysis.

All statistical analyses and graphs were performed in R^38^ using the following packages: ‘ade4’^39^, ‘adegenet’^40^, ‘car’^41^, ‘dplyr’^42^, ‘FSA’^43^, ‘ggplot2’^44^, ‘ggrain’^45^, ‘PMCMRplus’^46^, and ‘tidyr’^47^.

### Spore germination and gametophyte development of hybrid

To verify the spore germination ability of several hybrid samples, spore cultures were conducted. The sporangia were dried to release the spores, which were then transferred to distilled water. The spores were sterilized in a 1% NaClO solution for 5 min and then rinsed with distilled water. The sterilized spores were cultured on 1/4 MS medium at 25℃ with a 16 h/8 h light cycle. Subsequently, the germination of spores and growth of gametophytes were observed using a zoom stereo microscope (Optinity KS-200, Seongnam, Korea).

## Results

### Validation of genotypes and evaluation of the accuracy of CAPS markers

The *rbcL* product was 1,266 bp in length, and a total of five *rbcL* haplotypes were identified (Supplementary Table 2). Of these, four haplotypes matched those of *rbcL* I, I’, II, and III, as provided by Ebihara, et al.^12^, in 47 samples for which genome size analysis and ploidy level determination were possible. The frequencies of these haplotypes were: haplotype I (25.5%), haplotype I’ (21.3%), haplotype II (44.7%), and haplotype III (6.4%). Notably, haplotype III was found only in the triploid *γ*αβ genotype. The remaining *rbcL* haplotype, found in just one sample (CBNU2023-0054), differed from types I and I’ by one SNP each and was designated as type I’’. It was common to find different *rbcL* haplotypes among individuals growing side by side at the same collection site. Even in the case of two samples with identical nuclear *GapCp* sequences, identified through cloning, different *rbcL* haplotypes were observed (CBNU2019-0082 and CBNU2023-0011).

In the nuclear *GapCp* genotype analysis (Supplementary Table 2), the product length was 568–570 bp, and a total of 14 sequences were identified. Among these, seven sequences were categorized as the A genotype. Four sequences matched A1 (AB196370), A2 (AB196371), A5 (AB196374), and A13 (AB196382), as reported by Ebihara, et al.^12^. However, the remaining three sequences did not match any previously reported A genotypes and were designated AK1, AK2, and AK3 (Supplementary Table 3). Additionally, four sequences were categorized as the B genotype. Three matched B1 (AB196392), B2 (AB196393), and B3 (AB196394) as suggested by Ebihara, et al.^12^, while one, designated BK1, did not match any known B genotype (Supplementary Table 3). Furthermore, three sequences were identified as the C genotype, matching C1 (AB196397), C2 (AB196398), and C7 (AB196403).

The CAPS experiment for the nuclear *GapCp* identified five distinct genotypes (Fig. 2B). Non-hybrid individuals with genotypes AA/AAA and BB/BBB exhibited a single band pattern, while hybrid individuals displayed two or three bands. Cloning validations for each nuclear genotype confirmed the CAPS results. Each chloroplast haplotype (I, II, and III) corresponded to the nuclear genotypes (A, B, and C), respectively.

Among the 47 analyzed samples of *Vandenboschia* (Supplementary Table 2), the non-hybrid αα and ααα type were rare, with only four samples (9%) found in a southern valley of Jeju Island. Another non-hybrid group, the ββ and βββ types, comprised 10 samples (21%) found on both the Ulleung and Jeju Islands, with diploids being relatively frequent on Ulleung Island. Triploid hybrids were the most prevalent, comprising 29 samples (62%). These included 13 *α*αβ/*α*ββ/*α*β* types, 11 *β*αα/*β*βα/*β*α* types, 3 *γ*αβ types, and 2 *α*αγ types. These triploid hybrids containing α and β genomes were distributed across various regions. Tetraploid *α*αβγ or *α*ββγ types were rarely detected, with only a few occurrences on Jeju Island found in small and isolated populations.

### Confirmation of ploidy level and genome size

**Fig. 4:**
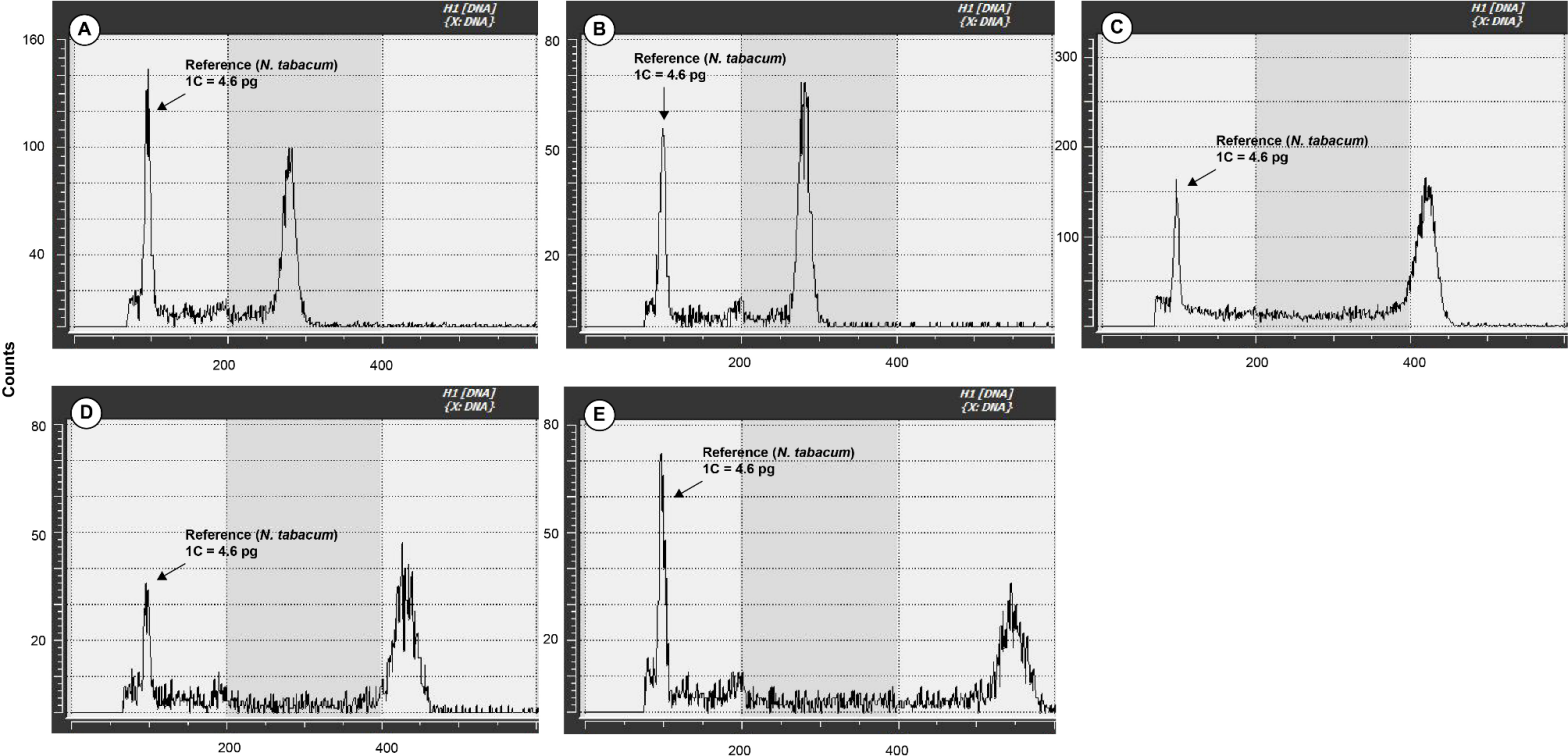
Examples of genome size measurement for the genus *Vandenboschia* in Korea. (**A**) Diploid *V. kalamocarpa* (CBNU2023-0009). **(B)** Diploid *V. nipponica* (CBNU2019-0086). (**C**) Triploid *V*. ×*stenosiphon* (CBNU2023-0016). (**D**) Triploid *V. ×quelpaertensis* (CBNU2020-0131). (**E**) Tetraploid *V. kalamocarpa* × *V. nipponica* × *V. striata* (CBNU2018-0377).

**Fig. 5:**
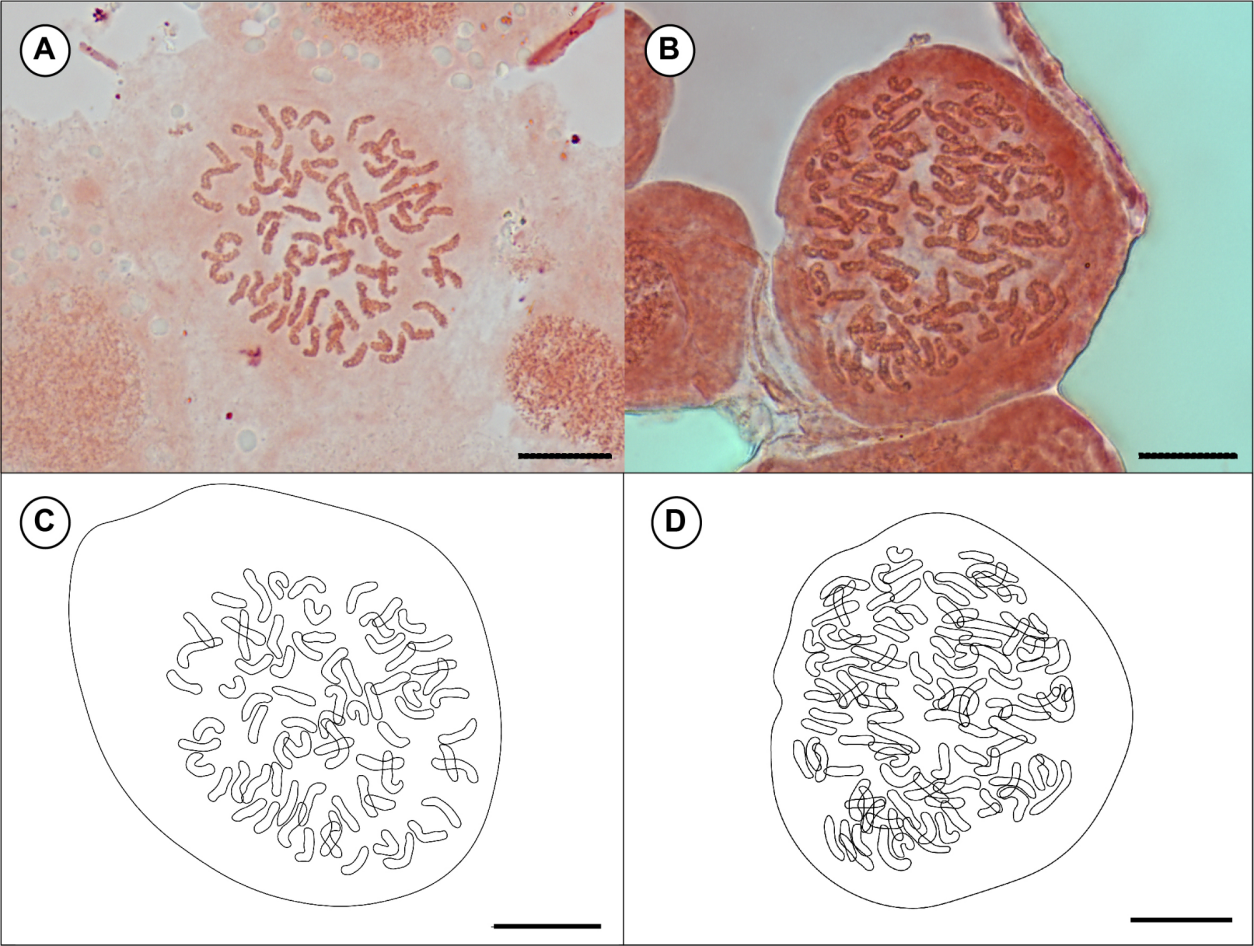
Mitotic chromosome counts (**A**, **B**) and explanatory illustrations (**C**, **D**) of *Vandenboschia* species. **A**, **C.** Diploid *V. nipponica* (2n = 2x = 72). **B**,** D.** Triploid *V. kalamocarpa* × *V. nipponica* × *V. striata* (2n = 3x = 108). Scale bar = 10 μm.

The genome sizes of *Vandenboschia* individuals were categorized into three distinct groups: 12.83 to 13.81 pg, 18.7 to 20.74 pg, and 25.26 to 26.42 pg (Fig. 4, Supplementary Table 2). The first two groups were confirmed as diploid (2n = 2x = 72) and triploid (2n = 3x = 108), respectively, through chromosome counting in somatic cells (Fig. 5). Unfortunately, it was not possible to observe and count the mitotic metaphase chromosome of the largest genome sized group at this time, but it was suggested to be tetraploids based on the comparison of genome size.

Triploids were frequently observed across the populations, whereas diploids and tetraploids were confined to specific populations (Supplementary Table 2). The average genome sizes were as follows: diploids averaged 13.29 ± 0.32 pg, triploids averaged 19.54 ± 0.50 pg, and tetraploids averaged 25.66 ± 0.54 pg. Among non-hybrid diploids, the αα genotype had an average genome size of 13.08 ± 0.07 pg, while the slightly larger ββ genotype averaged 13.47 ± 0.31 pg. Triploids showed average genome sizes of 19.05 ± 0.08 pg for the ααα genotype and 19.61 ± 0.35 pg for the βββ genotype. The triploid hybrids exhibited the following average genome sizes: 19.43 ± 0.44 pg for *α*αβ/*α*ββ/α*β/*β*αα/*β*βα/*β*α*, 19.85 ± 0.26 pg for *α*αγ, and 20.45 ± 0.22 pg for *α*βγ, which was the largest among the triploids. The tetraploid hybrid *α*αβγ/*α*ββγ averaged 25.66 ± 0.54 pg. Only one diploid *α*β (CBNU2018-0273) was identified, with a genome size of 12.83 pg.

### Comparison of morphological traits across each genotype

Ten morphological traits were compared across different genotypes (Table 1), and their measurement values are detailed in Supplementary Fig. 1 and Supplementary Table 4. The RD ranged from 0.31 to 0.67 mm, with average values varying between 0.41 mm (G-2) and 0.65 mm (G-4). Based on the ANOVA results (Supplementary Table 5), G-4 exhibited a significantly larger RD compared to other groups (adjusted p-value < 0.001), while no significant differences were found among G-1, G-2, and G-3 (adjusted p-value > 0.5).

Six leaf traits (SL, RL, PL, PW, and PN) exhibited similar patterns of morphological distribution to RD. G-4 differed significantly from G-1 and G-2 (adjusted p-value < 0.01). However, G-1 was not significantly different from G-2 (adjusted p-value > 0.05). Notably, SL was significantly greater in G-4 compared to G-1 (*p* = 0.004), G-2 (*p* < 0.001), and G-3 (*p* = 0.013). RL exhibited significant differences between G-1 and G-3 (*p* = 0.022), between G-1 and G-4 (*p* < 0.001), and between G-2 and G-4 (*p* = 0.005). PL was significantly larger in G-4 than in G-1 (*p* = 0.007), G-2 (*p* < 0.001), and G-3 (*p* = 0.044). PW showed significant differences between G-4 and G-1, G-2, and G-3 (*p* < 0.001). PN showed significant differences between G-4 and G-1 (*p* < 0.001), G-2 (*p* = 0.002), and G-3 (*p* = 0.002). The LW of G-4 was significantly different from all other groups (adjusted p-value < 0.001), but there were no significant differences among G-1, G-2, and G-3 (adjusted p-value > 0.05).

ANOVA revealed no significant difference in IW among groups (adjusted p-value > 0.1) due to overlapping ranges. IL of G-2 showed significant differences compared to G-3 and G-4 (adjusted p-value < 0.001). These two involucre traits exhibited lower resolution power compared to the leaf traits. However, the width-to-length ratio of the involucre showed significant differences among the groups (adjusted p-value < 0.01), except between G-1 and G-3 (adjusted p-value > 0.5).

### Discriminant analysis of principal components (DAPC)

The number of principal components was determined to be three, explaining at least 90% of the variance. Based on this number of principal components, the optimal number of discriminant functions was determined to be three using cross-validation. The proportion of variance explained by PCA was 93.6%. The first two linear discriminants (LDs) distinguished G-2 and G-4 independently, but G-1 and G-3 were not distinguished. The most contributive original variable was the width-to-length ratio of the involucre to LD1 and rhizome diameter (RD) to LD2.

**Fig. 6:**
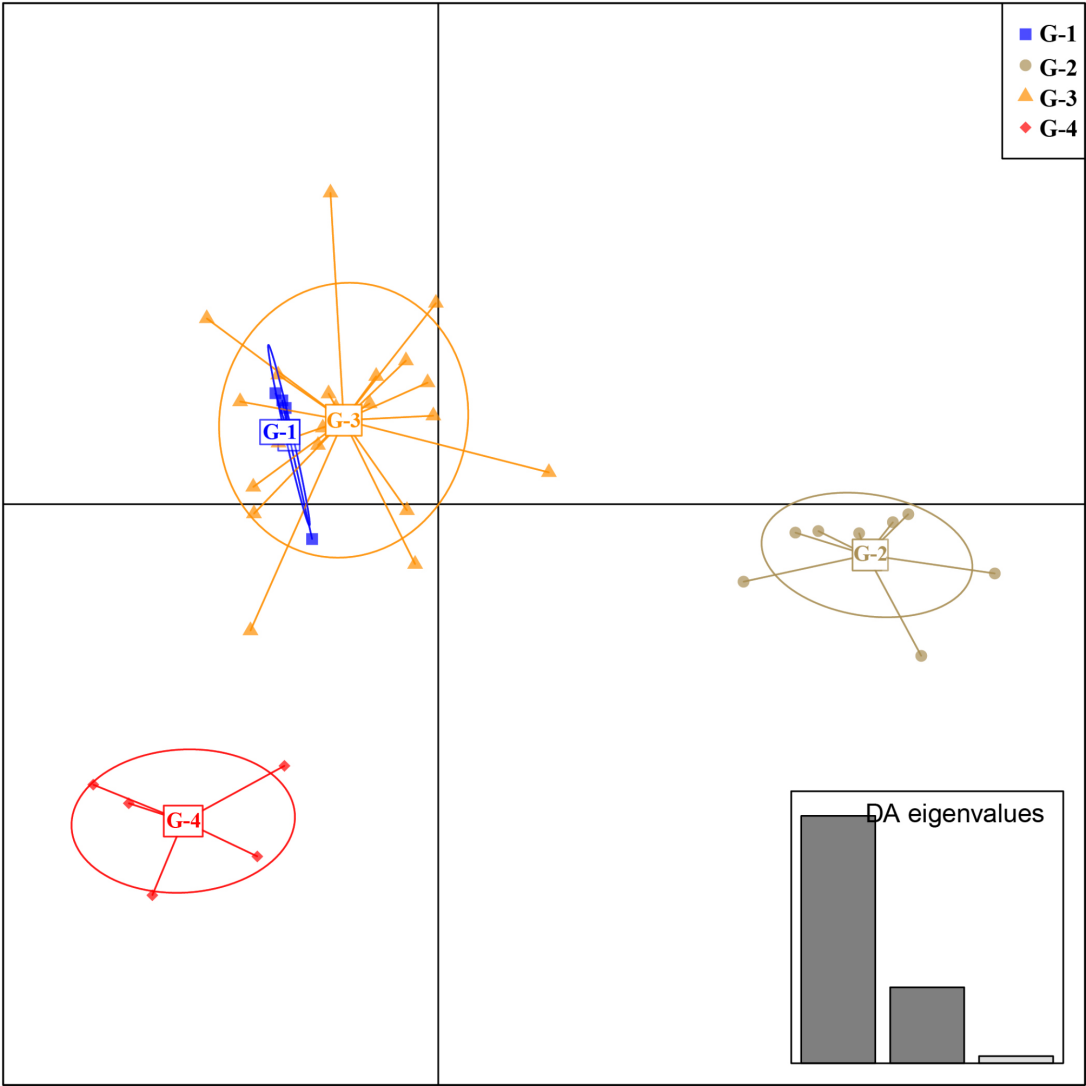
Result of discriminant analysis of principal components. The genotypes for each group are as follows: G-1: αα / ααα. G-2: ββ / βββ. G-3: *α*αβ, *α*ββ, *α*β* / *β*βα, *β*αα, *β*α*. G-4: *γ*βα / *α*αβγ, *α*ββγ. The genotype *α*αγ is excluded due to having only one measurable sample.

In DAPC using eight morphological traits, G-1 and G-4 were clearly distinguishable from other species (Fig. 6). The most significant variable for LD1 was the width-to-length ratio of the involucre, with significant differences observed in its averages across groups (Supplementary Table 5), except between G-1 and G-3. Ratios greater than 0.7 were found only in *V. nipponica*. Meanwhile, the most significant variable for LD2 was the RD (rhizome diameter). The average RD of the *V. kalamocarpa* × *V. nipponica* × *V. striata* hybrids was significantly greater than that of any other species, and it was unique to this combined hybrid with diameters over 0.6 mm.

### Spore germination of hybrid species

**Fig. 7:**
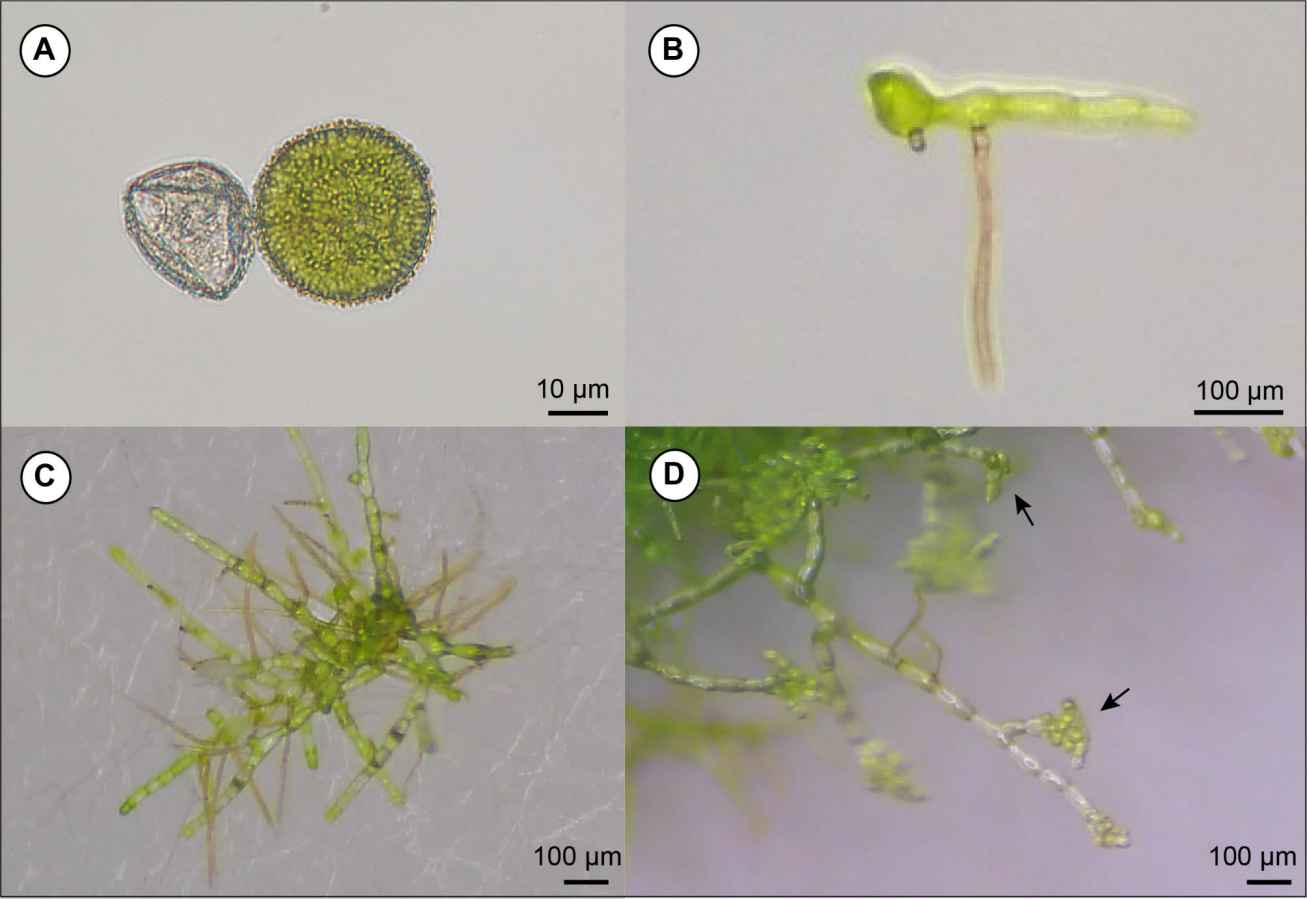
Result of the spore germination experiment of *V*. × *stenosiphon* over time. (**A**) Abortive spore (left) and normal spore (right). (**B**) Gametophyte 2 weeks after spore sowing. (**C**) Gametophyte 8 months after spore sowing. (**D**) Gemmae produced on gametophyte 14 months after spore sowing (indicated by arrows).

Normal chlorophyllous spores of the tetraploid *V. kalamocarpa* × *V. nipponica* × *V. striata* failed to germinate. However, it was confirmed that some spores from the triploid *V*. × *stenosiphon* successfully germinated into gametophytes, with rhizoid development observed 2 weeks after sowing (Fig. 7), following by subsequent branching and gemmae production occurring 10 months after sowing.

## Discussion

### Distribution patterns of the ***Vandenboschia radicans*** complex in Korea

**Fig. 8:**
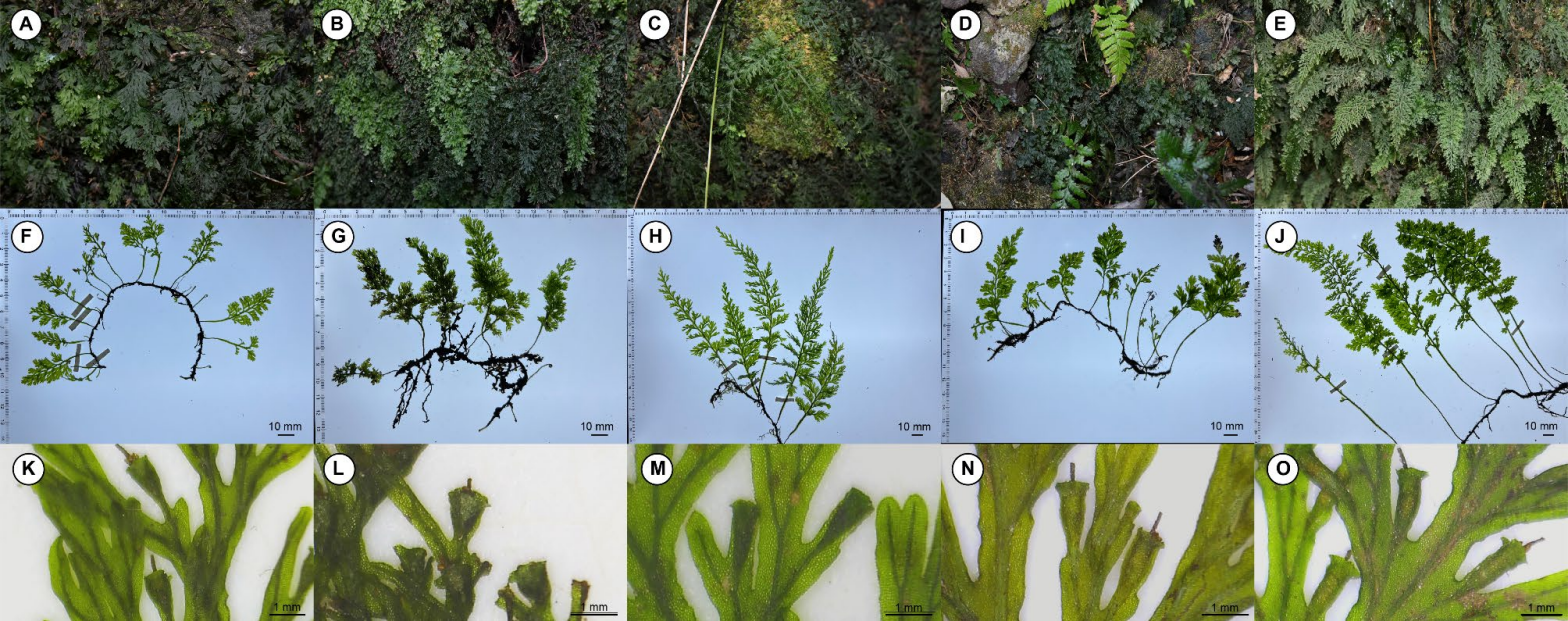
Morphological traits of Korean *Vandenboschia* species. **A–E.** Habitat views. (**A**) *V. kalamocarpa* (genotype: αα / ααα). (**B**) *V. nipponica* (genotype: ββ / βββ). (**C**) *V*. × *stenosiphon* (genotype: *α*β / *α*αβ, *α*ββ, *α*β* / *β*βα, *β*αα, *β*α*). (**D**) *V*. × *quelpaertensis* (genotype: *α*αγ). (**E**) *V. kalamocarpa* × *V. nipponica* × *V. striata* (genotype: *γ*βα / *α*αβγ, *α*ββγ). **F–J.** Leaf morphology. (**F**) *V. kalamocarpa*. (**G**) *V. nipponica*. (**H**) *V*. × *stenosiphon*. (**I**) *V*. × *quelpaertensis*. (**J**) *V. kalamocarpa* × *V. nipponica* × *V. striata*.). **K–O.** Involucre morphology. K. *V. kalamocarpa*. L. *V. nipponica*. M. *V*. × *stenosiphon*. N. *V*. × *quelpaertensis*. O. *V. kalamocarpa* × *V. nipponica* × *V. striata*.

This study represents the first comprehensive investigation of the *V. radicans* complex in Korea using morphological, cytological, and molecular approaches. As a result, five distinct taxa were finally identified (Fig. 8). Among these, the non-hybrid original species include *V. kalamocarpa* (αα/ααα) and *V. nipponica* (ββ/βββ), while the hybrid species comprise *V*. × *stenosiphon* (*α*β/*α*αβ/*α*ββ/*α*β*/*β*αα/*β*βα/*β*α*), *V.* × *quelpaertensis* (*α*αγ), and *V. kalamocarpa* × *V. nipponica* × *V. striata* (*γ*αβ/*α*αβγ/*α*ββγ).

Previously, many specimens deposited in Korean herbaria were identified as *V. kalamocarpa* and *V. nipponica*; however, these original species were not found at the same collection sites during our re-examination. Specifically, most *V. nipponica* specimens in herbaria had an IW: IL (involucre width-to-length) ratio of less than 0.6. Furthermore, given the restricted distribution of the original species identified in this study, it appears that *V*. ×*stenosiphon* (*α*β/*α*αβ/*α*ββ/*α*β*/*β*αα/*β*βα/*β*α*) has often been misidentified as *V. kalamocarpa* and *V. nipponica* due to their morphological similarities.

The detailed discussions regarding the five species confirmed in this study are as follows.

***Vandenboschia kalamocarpa*** *V. kalamocarpa* has traditionally been recognized by its “medium” sized flat leaf blades^12^. Although it was previously thought to be relatively common on Jeju Island^24–27^, our study found only a few individuals present near stream water in a valley in the southern part of Jeju Island (Fig. 1).

Contrary to the previous recognition of having “medium” sized leaves, the leaf size based on the stipe and rachis length of *V. kalamocarpa* is the smallest among the groups. It is notably smaller than that of *V. nipponica*, although this difference is not statistically significant (Supplementary Table 5). While the leaf blades tend to be flat overall as described by Ebihara^20^, some individuals exhibit a slight three-dimensional appearance due to the wavy wings of the rachis.

All morphological traits of *V. kalamocarpa* fall within the range of the hybrid *V*. × *stenosiphon*, making it difficult to distinguish between the two species based solely on morphological traits. Both diploid and triploid individuals of this species have been observed coexisting in the same habitat. In Japan, *V. kalamocarpa* is restricted to the warmer eastern regions, with diploids being rarer than triploids^19,28^. Our findings on Jeju Island are consistent with the case of Japanese *V. kalamocarpa*, as it is found only in the warmer southern regions of its distribution areas.

However, the Korean *V. kalamocarpa* does not appear to directly match the two αα genotypes reported from Japan (Supplementary Table 6). In fact, the frond size of the Korean *V. kalamocarpa* falls within the lower range of Japanese *V. kalamocarpa* and overlaps with the larger range of Japanese *V. subclatrata*^12^. Although this study is limited by the small sample size, further research comparing the two αα genotypes in Japan is necessary to better understand *V. kalamocarpa* in Korea.

***Vandenboschia Nipponica*** *V. nipponica* is recognized for its “small” leaf size and three-dimensional leaf structure. Previously, it was reported to be distributed on Ulleung and Jeju Islands^24–27^. However, the study by Ebihara, et al.^12^ on the *Vandenboschia radicans* complex suggested that *V. nipponica* is endemic to Japan, based on new molecular evidence^19,28^.

Interestingly, our study has conclusively confirmed its presence on Ulleung and Jeju Islands in Korea. In Japan, *V. nipponica* is found at higher latitudes than *V. kalamocarpa* and is primarily distributed in the western regions. Similarly, in Korea, it is distributed in the Nari basin of Ulleung Island and in the northern mountainous regions of Jeju Island, while *V. kalamocarpa* is found only in the southern valley of Jeju Island.

The key morphological traits for identifying *V. nipponica* in Korea, namely its small leaf size and three-dimensional appearance, are also observed in some individuals of *V*. × *stenosiphon*. Consequently, many specimens previously identified as *V. nipponica* on Jeju Island appear to have been misidentified and are actually *V*. × *stenosiphon*. This misidentification arises because *V. nipponica* (ββ/βββ) has a limited distribution, with small populations in specific areas. However, the width-to-length ratio of the involucre allows for clear differentiation of *V. nipponica* from other species without overlap. Therefore, we suggest this ratio as the key morphological trait for accurately identifying *V. nipponica*.

***Vandenboschia × stenosiphon*** This hybrid has a much wider distribution in Korea compared to its parental species, *V. kalamocarpa* and *V. nipponica* (Fig. 1), similar to the distribution pattern of Japanese *Vandenboschia* × *stenosiphon*^12,19,48^. Morphological traits of this hybrid range between those of *V. kalamocarpa* and *V. nipponica*, except for the traits of the involucre (Table 1; Fig. 8). Certain traits also overlap with *V. kalamocarpa* × *V. nipponica* × *V. striata*. The high morphological variation found in this hybrid has likely contributed to its misidentification within the *V. radicans* complex.

Within this species, both diploid and triploid hybrids were confirmed. Diploid hybrids were found in a single population on Jeju Island, similar to their distribution in Japan^19^. It is assumed that this diploid hybrid likely arises directly from diploid parents due to the restricted distribution and small population size of the two parental species on Jeju Island and the simplicity of hybridization within the complex. However, applying this hypothesis to the Japanese species is challenging given their distribution. Specifically, in Japan, diploid *V. kalamocarpa* mainly occurs along the southern Pacific coast, while diploid *V. nipponica* is found along the northern coast, making the likelihood of fertilization between these parental species very low.

The widespread distribution of triploid hybrids is particularly interesting. In Japan, this hybrid was suggested to be sterile^19^, and reproduction by apogamy was doubted because the high genetic variation in these triploid hybrids suggested sexual reproduction rather than clonal propagation^12^. The authors proposed that the wide and abundant distribution of hybrids, including triploid *V. × stenosiphon*, is a relic of hybridization events between fertile races in the past. This hypothesis is supported by the general understanding that hybrids are more resistant to environmental stresses than their original parents^49^. However, if extreme environmental stresses that cause population destruction accumulate over time, the number of populations might be reduced. Vegetative reproduction can generally increase the population size but typically does not increase the number of populations or their distribution. Therefore, solely vegetative reproduction is unlikely to sustain such widespread and abundant distributions unless the fertile races have vanished recently and abruptly.

The results of this study support the hypothesis proposed by Ebihara, et al.^12^ regarding this hybrid. A few normal chlorophyllous spores were found in the triploid *V.* × *stenosiphon*, and they successfully developed into gametophytes (Fig. 7). Some of the germinated gametophytes survived for over one year, and gemmae, which are vegetative reproductive organs, were observed (Fig. 7-D). Although we did not observe the development of sexual organs or confirm the ploidy levels of the gametophytes, these results suggest the possibility of new individuals arising through apogamy or sexual reproduction.

***Vandenboschia × quelpaertensis*** This hybrid species is a cross between *V. kalamocarpa* and *V. striata*, the latter of which has not yet been found in Korea. Although the type locality of this hybrid is Jeju Island^21^, it had been absent from Korean flora until recently. The scientific name of this species was revised to *Vandenboschia* × *quelpaertensis* (Nakai) Ebihara, comb. nov. based on its ploidy level and genotype^19^. Subsequently, *V.* × *quelpaertensis*, which exhibits large fronds, was reintroduced into the Korean fern flora^27^. However, our findings indicate that *V.* × *quelpaertensis* is very rare, and in most cases, the individuals with large fronds were identified as either *V*. × *stenosiphon* or *V. kalamocarpa* × *V. nipponica* × *V. striata*.

As only one individual of this species was found in the present study, we could not strictly describe the morphological traits of this hybrid. However, all traits of this individual fall within the range of *V*. × *stenosiphon* or *V. kalamocarpa* × *V. nipponica* × *V. striata*. Further comprehensive sampling is necessary to better understand and describe this hybrid in Korea.

***Vandenboschia kalamocarpa × Vandenboschia nipponica × Vandenboschia striata*** This hybrid, involving three original species, has not been previously recorded in Korea up to dates. It was discovered in two locations on Jeju Island in the present study. The first location was a deep pit in the northeastern part of Jeju Island, where both triploid and tetraploid individuals were identified alongside *V. × stenosiphon*. The second location was within a forest next to a waterfall, where only one individual was found. This specimen appeared to be young due to its small leaf without an involucre, but it was confirmed to be a tetraploid.

Morphologically, *V. kalamocarpa* × *V. nipponica* × *V. striata*, with large fronds, closely resembles *V. × stenosiphon* and *V.* × *quelpaertensis*. However, this hybrid has the thickest rhizome and the smallest width-to-length ratio of the involucre among the groups, with significant differences (Supplementary Table 5). Therefore, the key morphological traits for identifying this hybrid species should primarily consider the rhizome diameter and the width-to-length ratio of the involucre.

Ebihara et al. (2009b) described this hybrid as sterile; however, the presence of normal-shaped spores with chlorophylls in this hybrid does not rule out the possibility of generating gametophytes similar to the triploid *V. × stenosiphon*. In contrast to triploid *V. × stenosiphon*, the number of individuals and the distribution of this hybrid are very small and restricted. Therefore, although propagation by apogamy is possible, it may be limited.

Furthermore, the triploid of this hybrid is consistently the *γ*αβ genotype, whereas the tetraploid exhibits two different genotypes: *α*αβγ and *α*ββγ. The *γ*αβ genotype may produce unreduced gametophytes capable of forming sperm cells, which can fertilize *α*-type egg cells to generate the *α*αβγ genotype. In Jeju Island, both *α*α and *γ*αβ genotypes have been identified despite their remote locations. However, the *α*ββγ genotype possesses more complexity due to its maternal *α* genotype. The possible combinations for the *α*ββγ genotype are *α*β (egg cell) × βγ (sperm cell), *α*ββ (egg cell) × γ (sperm cell), *α*γ (egg cell) × ββ (sperm cell), *α*βγ (egg cell) × β (sperm cell), and *α* (egg cell) × ββγ (sperm cell). Based on our observations, it seems unlikely that these diverse combinations of the *α*ββγ genotype occur, given that *α*, *β*, *α*β, *β*β, and *α*ββ genotypes are likely to arise from both normal and abnormal meiosis. One possible hypothesis to explain this hybrid species is the translocation of counterpart gametes from other regions. For instance, in Japan, γ, αγ, and ββγ genotype gametophytes have been reported to arise through normal or abnormal meiosis^12^. Further comprehensive observations are required to verify this hypothesis.

## Electronic supplementary material

Below is the link to the electronic supplementary material.

Supplementary Material 1

Supplementary Material 2

Supplementary Material 3

## Data Availability

All of sequences generated from the present study are available in the NCBI database using the accession number described in Supplementary Table 3.
