# Supplementary figures and images for: Hybridization and diversity of the genus Vandenboschia in Korea insights from morphological, cytological, and genotype analyses
Source: Sci Rep. 2025 Jan 10;15:1619. doi: 10.1038/s41598-025-86000-3 (PMC11723990; doi:10.1038/s41598-025-86000-3)

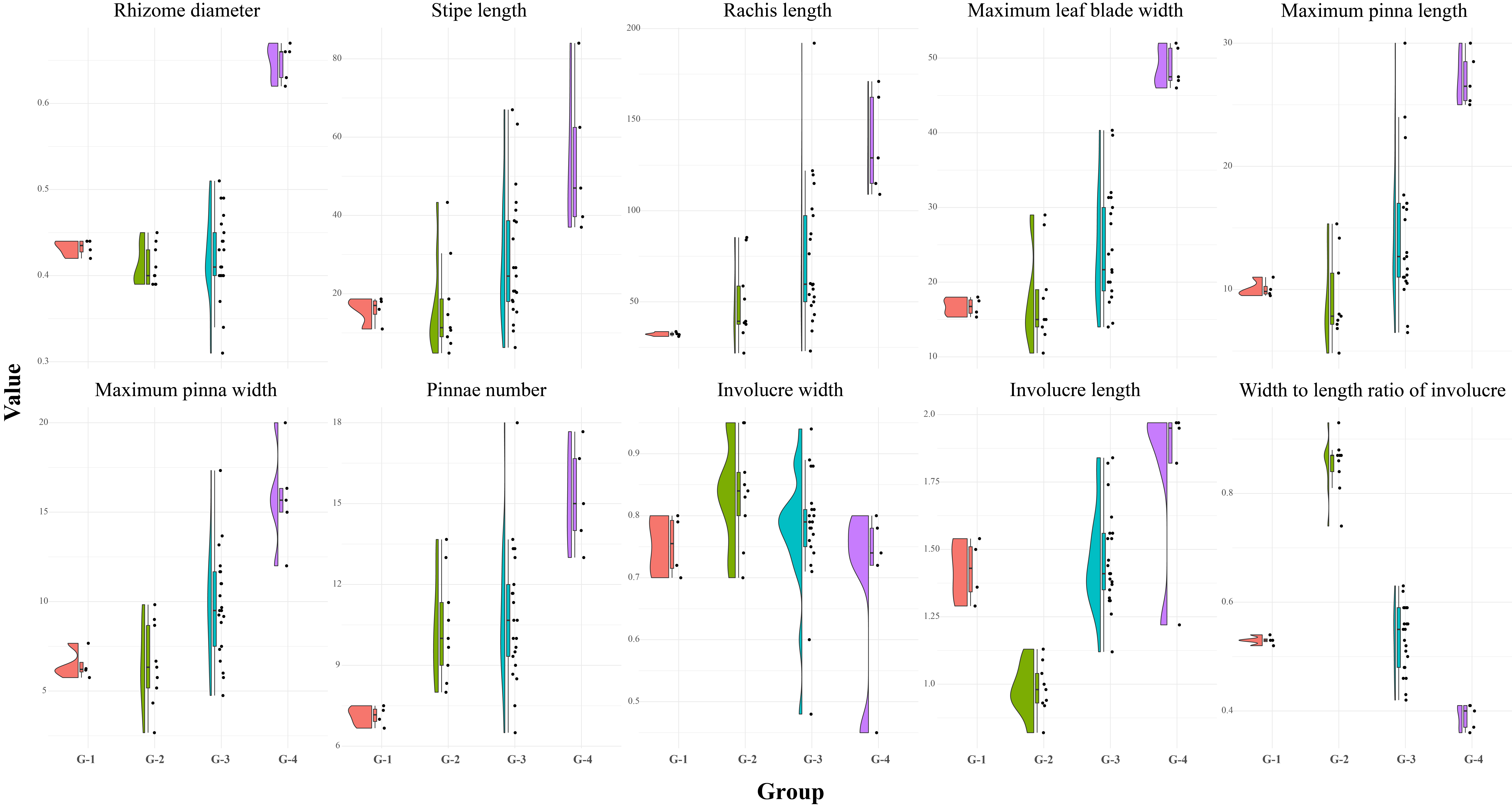

Supplement: Supplementary file 1 — Supplementary Material 1 [file 41598_2025_86000_MOESM1_ESM.jpg]
