# Supplementary material for: Hybridization and diversity of the genus Vandenboschia in Korea insights from morphological, cytological, and genotype analyses
Source: Sci Rep. 2025 Jan 10;15:1619. doi: 10.1038/s41598-025-86000-3 (PMC11723990; doi:10.1038/s41598-025-86000-3)
Supplement: Supplementary file 2 — Supplementary Material 2 [file 41598_2025_86000_MOESM2_ESM.docx]

**Supplementary materials**

**Supplementary Figure 1.** Box plots for the results of morphological trait measurements. The genotypes for each group are as follows: G-1: αα / ααα. G-2: ββ / βββ. G-3: ααβ, αββ, αβ* / ββα, βαα, βα*. G-4: γβα / ααβγ, αββγ . The genotype ααγ is excluded due to having only one measurable sample.

**Supplementary Table 1.** Changes in the classification for Korean *Vandenboschia* species recognized by morphological traits.

**Supplementary Table 2.** Collection site, genotype, ploidy, and genome size by sample voucher. Sixteen samples, which lack genome size data and ploidy determination, are marked as NA; their nuclear *GapCp* genotype is indicated within [ ].

**Supplementary Table 3.** GenBank accession numbers and sequence types of nuclear *GapCp* and chloroplast *rbcL* for each *Vandenboschia* sample. Each type follows the classification of Ebihara, et al. ^12^, and newly identified sequence types in this study are marked with an asterisk (*).

**Supplementary Table 4.** Statistical summary of morphological traits.

**Supplementary Table 5.** ANOVA test result for ten morphological traits.

**Supplementary Table 6.** Morphological traits of α-genotypes in the *Vandenboschia radicans* complex in Korea and Japan ^12^.
