# Supplementary material for: Hybridization and diversity of the genus Vandenboschia in Korea insights from morphological, cytological, and genotype analyses
Source: Sci Rep. 2025 Jan 10;15:1619. doi: 10.1038/s41598-025-86000-3 (PMC11723990; doi:10.1038/s41598-025-86000-3)
Supplement: Supplementary file 3 — Supplementary Material 3 [file 41598_2025_86000_MOESM3_ESM.docx]

**Supplementary Table 1.** Changes in the classification for Korean *Vandenboschia* species recognized by morphological traits.

| Morphological leaf traits  (Korean name) |  | Taxonomic treatment by researchers | | | | |
| --- | --- | --- | --- | --- | --- | --- |
|  | **Nakai (1952)** | **Chung (1955)** | **Park (1975)** | **Sun (2007);**  **Moon (2007)** | **Park et al. (2008)** | **Lee and Lee (2015)** |
| Medium, flat form  (Nu-un-goe-bul-i-kki) | *Trichomanes amabile* Nakai  *Trichomanes quelpaertense* Nakai  *Trichomanes stenosiphon* Christ | *Trichomanes orientalis* C. Chr. | *Vandenboschia radicans* var. *orientalis* (C.Chr.) H. Ito | *Crepidomanes radicans* (Sw.) K. Iwats. | *Lacosteopsis orientalis* (C. Chr.) Nakaike | *Vandenboschia kalamocarpa* (Hayata) Ebihara |
| Small, 3-dimensional form ( Nan-jang-i-i-kki) |  | - | *Vandenboschia amabilis* (Nakai) K. Iwats. | *Crepidomanes amabile* (Nakai) K. Iwats. | *Lacosteopsis orientalis* var. *abbreviata* (C. Chr.) Nakaike | *Vandenboschia nipponica* (Nakai) Ebihara |
| Hybrid species  (Je-ju-geo-bul-i-kki) |  | - | - | - | - | *Vandenboschia* ×*stenosiphon* (Christ) Copel. |
| Hybrid species  (Keun-geo-bul-i-kki) |  | - | - | - | - | *Vandenboschia* ×*quelpaertensis* (Nakai) Ebihara |

**Supplementary Table 2.** Collection site, genotype, ploidy, and genome size by sample voucher. Sixteen samples, which lack genome size data and ploidy determination, are marked as NA; their nuclear *GapCp* genotype is indicated within [ ]. If the nuclear genotype was identified only by CAPS without cloning, an asterisk (*) was placed next to the nuclear genotype.

| Locality | Voucher | Species | Nuclear *GapCp* type | Chloroplast *rbcL* type | Genotype | Ploidy | Genome size |
| --- | --- | --- | --- | --- | --- | --- | --- |
| Nari, Is. Ulleung | CBNU2019-0029 | *V*. ×*stenosiphon* | A_1_*B_1_ | Ⅱ | βα* | 3x | 19.71 |
|  | CBNU2019-0032 | *V. nipponica* | B_1_B_*_B_3_ | Ⅱ | βββ | 3x | 19.23 |
|  | CBNU2022-0059 | *V*. ×*stenosiphon* | A_1_*B_1_ | Ⅱ | βα* | 3x | 19.14 |
|  | CBNU2022-0061 | *V. nipponica* | B_1_B_2_ | Ⅱ | ββ | 2x | 13.37 |
|  | CBNU2022-0064 | *V. nipponica* | B_1_B_1_ | Ⅱ | ββ | 2x | 13.55 |
|  | CBNU2022-0066 | *V. nipponica* | B_1_B_1_ | Ⅱ | ββ | 2x | 13.70 |
|  | CBNU2022-0069 | *V. nipponica* | B_1_B_3_ | Ⅱ | ββ | 2x | 13.81 |
|  | CBNU2022-0074 | *V. nipponica* | B_1_B_*_B_3_ | Ⅱ | βββ | 3x | 19.69 |
| Hyodoncheon, Seogwipo-si, Is. Jeju | CBNU2020-0128 | *V*. ×*quelpaertensis* | A_1_A_2_C_7_ | Ⅰ | ααγ | 3x | 19.59 |
|  | CBNU2020-0130 | *V*. ×*stenosiphon* | A_2_*B_1_ | Ⅰ | αβ* | 3x | 19.44 |
|  | CBNU2020-0131 | *V*. ×*quelpaertensis* | A_1_A_2_C_7_ | Ⅰ’ | ααγ | 3x | 20.11 |
|  | CBNU2020-0147 | *V*. ×*stenosiphon* | A_12_A_K1_B_1_ | Ⅰ’ | ααβ | 3x | 18.87 |
| Sanghyodong, Seogwipo-si, Is. Jeju | CBNU2023-0054 | *V. kalamocarpa* | A_1_A_1_ | Ⅰ’’ | αα | 2x | 13.01 |
| Cheonjiyeon, Seogwipo-si, Is. Jeju | CBNU2023-0006 | *V. kalamocarpa* × *V. nipponica* × *V. striata* | A_2_A_K3_B_1_C_2_ | Ⅰ’ | ααβγ | 4x | 26.42 |
| Donnaeko, Seogwipo-si, Is. Jeju | CBNU2018-0156 | *V. kalamocarpa* | [A_*_] | Ⅰ | NA | NA | NA |
|  | CBNU2019-0049 | *Vandenboschia* sp. | [A_*_/B_*_] | Ⅰ’ | NA | NA | NA |
|  | CBNU2019-0054 | *V. kalamocarpa* | [A_*_] | Ⅰ | NA | NA | NA |
|  | CBNU2019-0057 | *V. kalamocarpa* | A_1_A_1_A_1_ | Ⅰ | ααα | 3x | 19.13 |
|  | CBNU2020-0016 | *Vandenboschia* sp. | [A_*_/B_*_] | Ⅰ | NA | NA | NA |
|  | CBNU2023-0007 | *V. kalamocarpa* | A_1_A_1_A_1_ | Ⅰ | ααα | 3x | 18.97 |
|  | CBNU2023-0009 | *V. kalamocarpa* | A_1_A_2_ | Ⅰ | αα | 2x | 13.15 |
|  | KHB1079907 | *Vandenboschia* sp. | [A_2_/C_2_/C_3_] | Ⅲ | NA | NA | NA |
| Seongeup-ri, Seogwipo-si, Is. Jeju | CBNU2018-0270 | *Vandenboschia* sp. | [A_*_/B_*_] | Ⅱ | NA | NA | NA |
|  | CBNU2018-0353 | *V*. ×*stenosiphon* | A_1_A_5_B_K1_ | Ⅰ’ | ααβ | 3x | 19.03 |
|  | CBNU2018-0354 | *Vandenboschia* sp. | [A_*_/B_*_] | Ⅱ | NA | NA | NA |
| Saerani, Jeju-si, Is. Jeju | CBNU2023-0041 | *V. nipponica* | B_1_B_1_B_1_ | Ⅱ | βββ | 3x | 20.20 |
|  | CBNU2023-0043 | *V*. ×*stenosiphon* | A_1_*B_1_ | Ⅱ | βα* | 3x | 20.10 |
|  | CBNU2023-0045 | *V*. ×*stenosiphon* | A_1_B_1_B_K1_ | Ⅱ | ββα | 3x | 19.95 |
| Songdangri, Jeju-si, Is. Jeju | CBNU2018-0373 | *V*. ×*stenosiphon* | A_1_A_K2_B_1_ | Ⅰ’ | ααβ | 3x | 19.50 |
|  | CBNU2018-0375 | *V. kalamocarpa* × *V. nipponica* × *V. striata* | A_K2_B_1_B_K1_C_1_ | Ⅰ’ | αββγ | 4x | 25.29 |
|  | CBNU2018-0376 | *V*. ×*stenosiphon* | A_K1_B_1_B_3_ | Ⅰ’ | αββ | 3x | 18.89 |
|  | CBNU2018-0377 | *V. kalamocarpa* × *V. nipponica* × *V. striata* | A_1_A_2_B_1_C_2_ | Ⅰ’ | ααβγ | 4x | 25.26 |
|  | CBNU2022-0008 | *V. kalamocarpa* × *V. nipponica* × *V. striata* | A_1_B_K1_C_1_ | Ⅲ | γαβ | 3x | 20.39 |
|  | CBNU2023-0012 | *V. kalamocarpa* × *V. nipponica* × *V. striata* | A_1_B_K1_C_1_ | Ⅲ | γαβ | 3x | 20.74 |
|  | CBNU2023-0013 | *V*. ×*stenosiphon* | A_1_A_2_B_1_ | Ⅰ | ααβ | 3x | 19.86 |
|  | CBNU2023-0014 | *V. kalamocarpa* × *V. nipponica* × *V. striata* | A_1_B_K1_C_1_ | Ⅲ | γαβ | 3x | 20.21 |
|  | CBNU2023-0015 | *V*. ×*stenosiphon* | A_2_A_5_B_1_ | Ⅱ | βαα | 3x | 19.80 |
|  | CBNU2023-0016 | *V*. ×*stenosiphon* | A_1_A_5_B_1_ | Ⅰ | ααβ | 3x | 19.80 |
| Nohyeongdong, Jeju-si, Is. Jeju | CBNU2019-0132 | *V*. ×*stenosiphon* | A_1_B_1_B_3_ | Ⅱ | ββα | 3x | 19.59 |
|  | CBNU2019-0133 | *Vandenboschia* sp. | [A_*_/B_*_] | Ⅱ | NA | NA | NA |
|  | CBNU2019-0134 | *V*. ×*stenosiphon* | A_1_*B_3_ | Ⅱ | βα* | 3x | 19.31 |
|  | CBNU2019-0135 | *V*. ×*stenosiphon* | A_1_*B_2_ | Ⅰ’ | αβ* | 3x | 19.57 |
|  | CBNU2023-0024 | *V. nipponica* | B_1_B_1_B_1_ | Ⅱ | βββ | 3x | 19.67 |
|  | CBNU2023-0025 | *V. nipponica* | B_1_B_1_B_1_ | Ⅱ | βββ | 3x | 19.26 |
| Gwangreong, Aewol, Jeju-si, Is. Jeju | CBNU2019-0076 | *V*. ×*stenosiphon* | A_1_A_2_B_1_ | Ⅱ | βαα | 3x | 19.18 |
| Yusuam, Aewol, Jeju-si, Is. Jeju | CBNU2019-0082 | *V*. ×*stenosiphon* | A_K1_B_1_B_3_ | Ⅰ’ | αββ | 3x | 19.44 |
|  | CBNU2019-0086 | *V. nipponica* | B_1_B_3_ | Ⅱ | ββ | 2x | 12.93 |
|  | CBNU2023-0010 | *V*. ×*stenosiphon* | A_K1_B_1_B_3_ | Ⅱ | ββα | 3x | 19.57 |
|  | CBNU2023-0011 | *V*. ×*stenosiphon* | A_1_B_1_B_3_ | Ⅰ | αββ | 3x | 20.42 |
| Tamna Valley, Jeju-si, Is. Jeju | CBNU2019-0172 | *V*. ×*stenosiphon* | A_1_*B_1_ | Ⅰ | αβ* | 3x | 19.50 |
|  | CBNU2019-0173 | *Vandenboschia* sp. | [A_*_/B_*_] | Ⅰ | NA | NA | NA |
|  | CBNU2019-0174 | *V*. ×*stenosiphon* | A_1_B_1_B_3_ | Ⅱ | ββα | 3x | 18.97 |
| Gyorae-ri, Jeju-si, Is. Jeju | CBNU2018-0160 | *Vandenboschia* sp. | [A_*_/B_*_] | Ⅱ | NA | NA | NA |
|  | CBNU2018-0161 | *Vandenboschia* sp. | [A_*_/B_*_] | Ⅱ | NA | NA | NA |
|  | CBNU2018-0359 | *Vandenboschia* sp. | [A_*_/B_*_] | Ⅰ | NA | NA | NA |
|  | CBNU2018-0362 | *Vandenboschia* sp. | [A_*_/B_*_] | Ⅱ | NA | NA | NA |
|  | CBNU2018-0365 | *V*. ×*stenosiphon* | A_2_B_1_B_3_ | Ⅱ | ββα | 3x | 18.82 |
| Mulchat, Gyorae-ri, Jeju-si, Is. Jeju | CBNU2018-0273 | *V*. ×*stenosiphon* | A_1_B_1_ | Ⅰ | αβ | 2x | 12.83 |
| Seonheulri, Jeju-si, Is. Jeju | CBNU2020-0154 | *V*. ×*stenosiphon* | A_2_A_13_B_1_ | Ⅰ | ααβ | 3x | 18.70 |
|  | CBNU2020-0155 | *V*. ×*stenosiphon* | A_2_A_13_B_1_ | Ⅰ | ααβ | 3x | 19.29 |
| Hallimeup, Jeju-si, Is. Jeju | KHB1038189 | *Vandenboschia* sp. | [A_2_/A_*_/C_3_] | Ⅰ | NA | NA | NA |
| Cheongsong-gun, Gyeongsangbuk-do | NIBRVP0000200566 | *Vandenboschia* sp. | [A_1_/B_1_] | Ⅱ | NA | NA | NA |
| Haenam-gun, Jeollanam-do | NIBRVP0000329371 | *Vandenboschia* sp. | [A_k1_/B_1_] | Ⅰ | NA | NA | NA |

**Supplementary Table 3.** GenBank accession numbers and sequence types of nuclear *GapCp* and chloroplast *rbcL* for each *Vandenboschia* sample. Each type follows the classification of Ebihara et al. (2005), and newly identified sequence types in this study are marked with an asterisk (*).

| Voucher | Species | Genotype | Nuclear *GapCp* | | Chloroplast *rbcL* | |
| --- | --- | --- | --- | --- | --- | --- |
|  |  |  | Accession no. | Sequence type | Accession no. | Sequence type |
| CBNU2019-0029 | *V*. ×*stenosiphon* | βα* | PQ557716  PQ557717 | A_1_  B_1_ | PQ557831 | Ⅱ |
| CBNU2019-0032 | *V. nipponica* | βββ | PQ557718  PQ557719 | B_1_  B_3_ | PQ557832 | Ⅱ |
| CBNU2022-0059 | *V*. ×*stenosiphon* | βα* | PQ557760  PQ557761 | A_1_  B_1_ | PQ557854 | Ⅱ |
| CBNU2022-0061 | *V. nipponica* | ββ | PQ557762  PQ557763 | B_1_  B_2_ | PQ557855 | Ⅱ |
| CBNU2022-0064 | *V. nipponica* | ββ | PQ557812 | B_1_ | PQ557856 | Ⅱ |
| CBNU2022-0066 | *V. nipponica* | ββ | PQ557813 | B_1_ | PQ557857 | Ⅱ |
| CBNU2022-0069 | *V. nipponica* | ββ | PQ557764  PQ557765 | B_1_  B_3_ | PQ557858 | Ⅱ |
| CBNU2022-0074 | *V. nipponica* | βββ | PQ557766  PQ557767 | B_1_  B_3_ | PQ557859 | Ⅱ |
| CBNU2020-0128 | *V*. ×*quelpaertensis* | ααγ | PQ557740  PQ557741  PQ557742 | A_1_  A_2_  C_7_ | PQ557847 | Ⅰ |
| CBNU2020-0130 | *V*. ×*stenosiphon* | αβ* | PQ557743  PQ557744 | A_2_  B_1_ | PQ557848 | Ⅰ |
| CBNU2020-0131 | *V*. ×*quelpaertensis* | ααγ | PQ557745  PQ557746  PQ557747 | A_1_  A_2_  C_7_ | PQ557849 | Ⅰ’ |
| CBNU2020-0147 | *V*. ×*stenosiphon* | ααβ | PQ557748  PQ557749  PQ557750 | A_12_  A_K1_*  B_1_ | PQ557850 | Ⅰ’ |
| CBNU2023-0054 | *V. kalamocarpa* | αα | PQ557816 | A_1_ | PQ557875 | Ⅰ’’* |
| CBNU2023-0006 | *V. kalamocarpa* × *V. nipponica* × *V. striata* | ααβγ | PQ557768  PQ557769  PQ557770  PQ557771 | A_2_  A_K3_*  B_1_  C_2_ | PQ557860 | Ⅰ’ |
| CBNU2018-0156 | NA | NA | - | - | PQ557817 | Ⅰ |
| CBNU2019-0049 | NA | NA | - | - | PQ557833 | Ⅰ’ |
| CBNU2019-0054 | NA | NA | - | - | PQ557834 | Ⅰ |
| CBNU2019-0057 | *V. kalamocarpa* | ααα | PQ557811 | A_1_ | PQ557835 | Ⅰ |
| CBNU2020-0016 | NA | NA | - |  | PQ557846 | Ⅰ |
| CBNU2023-0007 | *V. kalamocarpa* | ααα | PQ557772 | A_1_ | PQ557861 | Ⅰ |
| CBNU2023-0009 | *V. kalamocarpa* | αα | PQ557773  PQ557774 | A_1_  A_2_ | PQ557862 | Ⅰ |
| KHB1079907 | NA | NA | PQ557808  PQ557809  PQ557810 | A_2_  C_2_  C_3_ | PQ557879 | Ⅲ |
| CBNU2018-0270 | NA | NA | - | - | PQ557820 | Ⅱ |
| CBNU2018-0353 | *V*. ×*stenosiphon* | ααβ | PQ557696  PQ557697  PQ557698 | A_1_  A_5_  B_K1_* | PQ557822 | Ⅰ’ |
| CBNU2018-0354 | NA | NA | - | - | PQ557823 | Ⅱ |
| CBNU2023-0041 | *V. nipponica* | βββ | PQ557815 | B_1_ | PQ557872 | Ⅱ |
| CBNU2023-0043 | *V*. ×*stenosiphon* | βα* | PQ557797  PQ557798 | A_1_  B_1_ | PQ557873 | Ⅱ |
| CBNU2023-0045 | *V*. ×*stenosiphon* | ββα | PQ557799  PQ557800  PQ557801 | A_1_  B_1_  B_K1_* | PQ557874 | Ⅱ |
| CBNU2018-0373 | *V*. ×*stenosiphon* | ααβ | PQ557702  PQ557703  PQ557704 | A_1_  A_K2_*  B_1_ | PQ557827 | Ⅰ’ |
| CBNU2018-0375 | *V. kalamocarpa* × *V. nipponica* × *V. striata* | αββγ | PQ557705  PQ557706  PQ557707  PQ557708 | A_K2_*  B_1_  B_K1_*  C_1_ | PQ557828 | Ⅰ’ |
| CBNU2018-0376 | *V*. ×*stenosiphon* | αββ | PQ557709  PQ557710  PQ557711 | A_K1_*  B_1_  B_3_ | PQ557829 | Ⅰ’ |
| CBNU2018-0377 | *V. kalamocarpa* × *V. nipponica* × *V. striata* | ααβγ | PQ557712  PQ557713  PQ557714  PQ557715 | A_1_  A_2_  B_1_  C_2_ | PQ557830 | Ⅰ’ |
| CBNU2022-0008 | *V. kalamocarpa* × *V. nipponica* × *V. striata* | γαβ | PQ557757  PQ557758  PQ557759 | A_1_  B_K1_*  C_1_ | PQ557853 | Ⅲ |
| CBNU2023-0012 | *V. kalamocarpa* × *V. nipponica* × *V. striata* | γαβ | PQ557781  PQ557782  PQ557783 | A_1_  B_K1_*  C_1_ | PQ557865 | Ⅲ |
| CBNU2023-0013 | *V*. ×*stenosiphon* | ααβ | PQ557784  PQ557785  PQ557786 | A_1_  A_2_  B_1_ | PQ557866 | Ⅰ |
| CBNU2023-0014 | *V. kalamocarpa* × *V. nipponica* × *V. striata* | γαβ | PQ557787  PQ557788  PQ557789 | A_1_  B_K1_*  C_1_ | PQ557867 | Ⅲ |
| CBNU2023-0015 | *V*. ×*stenosiphon* | βαα | PQ557790  PQ557791  PQ557792 | A_2_  A_5_  B_1_ | PQ557868 | Ⅱ |
| CBNU2023-0016 | *V*. ×*stenosiphon* | ααβ | PQ557793  PQ557794  PQ557795 | A_1_  A_5_  B_1_ | PQ557869 | Ⅰ |
| CBNU2019-0132 | *V*. ×*stenosiphon* | ββα | PQ557728  PQ557729  PQ557730 | A_1_  B_1_  B_3_ | PQ557839 | Ⅱ |
| CBNU2019-0133 | NA | NA | - | - | PQ557840 | Ⅱ |
| CBNU2019-0134 | *V*. ×*stenosiphon* | βα* | PQ557731  PQ557732 | A_1_  B_3_ | PQ557841 | Ⅱ |
| CBNU2019-0135 | *V*. ×*stenosiphon* | αβ* | PQ557733  PQ557734 | A_1_  B_2_ | PQ557842 | Ⅰ’ |
| CBNU2023-0024 | *V. nipponica* | βββ | PQ557796 | B_1_ | PQ557870 | Ⅱ |
| CBNU2023-0025 | *V. nipponica* | βββ | PQ557814 | B_1_ | PQ557871 | Ⅱ |
| CBNU2019-0076 | *V*. ×*stenosiphon* | βαα | PQ557720  PQ557721  PQ557722 | A_1_  A_2_  B_1_ | PQ557836 | Ⅱ |
| CBNU2019-0082 | *V*. ×*stenosiphon* | αββ | PQ557723  PQ557724  PQ557725 | A_K1_*  B_1_  B_3_ | PQ557837 | Ⅰ’ |
| CBNU2019-0086 | *V. nipponica* | ββ | PQ557726  PQ557727 | B_1_  B_3_ | PQ557838 | Ⅱ |
| CBNU2023-0010 | *V*. ×*stenosiphon* | ββα | PQ557775  PQ557776  PQ557777 | A_K1_*  B_1_  B_3_ | PQ557863 | Ⅱ |
| CBNU2023-0011 | *V*. ×*stenosiphon* | αββ | PQ557778  PQ557779  PQ557780 | A_1_  B_1_  B_3_ | PQ557864 | Ⅰ |
| CBNU2019-0172 | *V*. ×*stenosiphon* | αβ* | PQ557735  PQ557736 | A_1_  B_1_ | PQ557843 | Ⅰ |
| CBNU2019-0173 | NA | NA | - | - | PQ557844 | Ⅰ |
| CBNU2019-0174 | *V*. ×*stenosiphon* | ββα | PQ557737  PQ557738  PQ557739 | A_1_  B_1_  B_3_ | PQ557845 | Ⅱ |
| CBNU2018-0160 | NA | NA | - | - | PQ557818 | Ⅱ |
| CBNU2018-0161 | NA | NA | - | - | PQ557819 | Ⅱ |
| CBNU2018-0359 | NA | NA | - | - | PQ557824 | Ⅰ |
| CBNU2018-0362 | NA | NA | - | - | PQ557825 | Ⅱ |
| CBNU2018-0365 | *V*. ×*stenosiphon* | ββα | PQ557699  PQ557700  PQ557701 | A_2_  B_1_  B_3_ | PQ557826 | Ⅱ |
| CBNU2018-0273 | *V*. ×*stenosiphon* | αβ | PQ557694  PQ557695 | A_1_  B_1_ | PQ557821 | Ⅰ |
| CBNU2020-0154 | *V*. ×*stenosiphon* | ααβ | PQ557751  PQ557752  PQ557753 | A_2_  A_13_  B_1_ | PQ557851 | Ⅰ |
| CBNU2020-0155 | *V*. ×*stenosiphon* | ααβ | PQ557754  PQ557755  PQ557756 | A_2_  A_13_  B_1_ | PQ557852 | Ⅰ |
| KHB1038189 | NA | NA | PQ557806  PQ557807 | A_2_  C_3_ | PQ557878 | Ⅰ |
| NIBRVP0000200566 | NA | NA | PQ557802  PQ557803 | A_1_  B_1_ | PQ557876 | Ⅱ |
| NIBRVP0000329371 | NA | NA | PQ557804  PQ557805 | A_k1_*  B_1_ | PQ557877 | Ⅰ |

**Supplementary Table 4.** Statistical summary of morphological traits.

| Variable | Individual statistics | | | | Group statistics | |
| --- | --- | --- | --- | --- | --- | --- |
|  | Min | Max | Average | SD | Min Average (Group) | Max Average (Group) |
| Rhizome | | | | | | |
| Rhizome diameter (mm) | 0.31 | 0.67 | 0.45 | 0.09 | 0.41 (G-2) | 0.65 (G-4) |
| Leaf | | | | | | |
| Stipe length (mm) | 4.83 | 84.00 | 28.05 | 18.84 | 15.92 (G-1) | 54.03 (G-4) |
| Rachis length (mm) | 21.83 | 192.00 | 72.83 | 42.96 | 32.29 (G-1) | 137.27 (G-4) |
| Maximum leaf blade width (mm) | 10.50 | 52.00 | 25.26 | 11.56 | 16.71 (G-1) | 48.77 (G-4) |
| Maximum pinna length (mm) | 4.83 | 30.00 | 14.44 | 7.05 | 9.22 (G-2) | 27.07 (G-4) |
| Maximum pinna width (mm) | 2.67 | 20.00 | 9.46 | 3.89 | 6.46 (G-1) | 15.80 (G-4) |
| Pinnae number | 6.50 | 18.00 | 10.94 | 2.95 | 7.13 (G-1) | 15.27 (G-4) |
| Involucre | | | | | | |
| Involucre width (mm) | 0.45 | 0.95 | 0.78 | 0.10 | 0.70 (G-4) | 0.84 (G-2) |
| Involucre length (mm) | 0.82 | 1.97 | 1.39 | 0.31 | 0.98 (G-1) | 1.79 (G-4) |
| Width to length ratio of involucre | 0.36 | 0.93 | 0.59 | 0.16 | 0.39 (G-4) | 0.85 (G-2) |

**Supplementary Table 5.** ANOVA test result for ten morphological traits.

| Normality = yes & Homogeneity of Variances= yes | | | | | | Normality = no | | | | |
| --- | --- | --- | --- | --- | --- | --- | --- | --- | --- | --- |
| Variable | Comparison (Group) | diff | lwr | upr | p-adj | Variable | Comparison (Group) | Z | P.unadj | P.adj |
| Rhizome diameter | G-2 - G-1 | -0.021 | -0.085 | 0.042 | 0.800 | Rachis length | G-1 - G-2 | -1.334 | 0.182 | 0.182 |
|  | G-3 - G-1 | -0.011 | -0.068 | 0.047 | 0.959 |  | G-1 - G-3 | -2.770 | 0.006 | 0.022 |
|  | G-4 - G-1 | 0.216 | 0.145 | 0.286 | 0.000 |  | G-2 - G-3 | -1.780 | 0.075 | 0.150 |
|  | G-3 - G-2 | 0.011 | -0.031 | 0.053 | 0.899 |  | G-1 - G-4 | -3.942 | 0.000 | 0.000 |
|  | G-4 - G-2 | 0.237 | 0.178 | 0.296 | 0.000 |  | G-2 - G-4 | -3.304 | 0.001 | 0.005 |
|  | G-4 - G-3 | 0.226 | 0.174 | 0.279 | 0.000 |  | G-3 - G-4 | -2.278 | 0.023 | 0.068 |
| Stipe length | G-2 - G-1 | 0.767 | -24.248 | 25.782 | 1.000 | Maximum pinna length | G-1 - G-2 | 0.107 | 0.914 | 0.914 |
|  | G-3 - G-1 | 13.126 | -9.584 | 35.836 | 0.415 |  | G-1 - G-3 | -1.615 | 0.106 | 0.213 |
|  | G-4 - G-1 | 38.117 | 10.192 | 66.041 | 0.004 |  | G-2 - G-3 | -2.373 | 0.018 | 0.053 |
|  | G-3 - G-2 | 12.359 | -4.226 | 28.944 | 0.204 |  | G-1 - G-4 | -3.201 | 0.001 | 0.007 |
|  | G-4 - G-2 | 37.350 | 14.131 | 60.568 | 0.001 |  | G-2 - G-4 | -3.966 | 0.000 | 0.000 |
|  | G-4 - G-3 | 24.991 | 4.276 | 45.705 | 0.013 |  | G-3 - G-4 | -2.545 | 0.011 | 0.044 |
| Maximum leaf blade width | G-2 - G-1 | 1.181 | -9.484 | 11.847 | 0.991 | Involucre width | G-1 - G-2 | -1.875 | 0.061 | 0.304 |
|  | G-3 - G-1 | 7.740 | -1.942 | 17.423 | 0.156 |  | G-1 - G-3 | -0.946 | 0.344 | 0.688 |
|  | G-4 - G-1 | 32.059 | 20.153 | 43.964 | 0.000 |  | G-2 - G-3 | 1.532 | 0.126 | 0.502 |
|  | G-3 - G-2 | 6.559 | -0.512 | 13.630 | 0.077 |  | G-1 - G-4 | 0.252 | 0.801 | 0.801 |
|  | G-4 - G-2 | 30.877 | 20.978 | 40.777 | 0.000 |  | G-2 - G-4 | 2.323 | 0.020 | 0.121 |
|  | G-4 - G-3 | 24.318 | 15.487 | 33.150 | 0.000 |  | G-3 - G-4 | 1.378 | 0.168 | 0.505 |
| Maximum pinna width | G-2 - G-1 | 0.031 | -4.355 | 4.417 | 1.000 | Involucre length | G-1 - G-2 | 2.357 | 0.018 | 0.074 |
|  | G-3 - G-1 | 3.338 | -0.644 | 7.320 | 0.127 |  | G-1 - G-3 | -0.331 | 0.740 | 0.740 |
|  | G-4 - G-1 | 9.340 | 4.444 | 14.236 | 0.000 |  | G-2 - G-3 | -4.008 | 0.000 | 0.000 |
|  | G-3 - G-2 | 3.307 | 0.399 | 6.215 | 0.021 |  | G-1 - G-4 | -1.393 | 0.164 | 0.327 |
|  | G-4 - G-2 | 9.309 | 5.238 | 13.380 | 0.000 |  | G-2 - G-4 | -4.214 | 0.000 | 0.000 |
|  | G-4 - G-3 | 6.002 | 2.370 | 9.634 | 0.000 |  | G-3 - G-4 | -1.515 | 0.130 | 0.389 |
| Pinnae number | G-2 - G-1 | 3.283 | -0.350 | 6.915 | 0.088 |  |  |  |  |  |
|  | G-3 - G-1 | 3.733 | 0.435 | 7.030 | 0.021 |  |  |  |  |  |
|  | G-4 - G-1 | 8.143 | 4.088 | 12.198 | 0.000 |  |  |  |  |  |
|  | G-3 - G-2 | 0.450 | -1.958 | 2.858 | 0.958 |  |  |  |  |  |
|  | G-4 - G-2 | 4.860 | 1.489 | 8.232 | 0.002 |  |  |  |  |  |
|  | G-4 - G-3 | 4.410 | 1.402 | 7.418 | 0.002 |  |  |  |  |  |
| Width to length ratio of involucre | G-2 - G-1 | 0.322 | 0.236 | 0.408 | 0.000 |  |  |  |  |  |
|  | G-3 - G-1 | 0.002 | -0.076 | 0.081 | 1.000 |  |  |  |  |  |
|  | G-4 - G-1 | -0.140 | -0.236 | -0.044 | 0.002 |  |  |  |  |  |
|  | G-3 - G-2 | -0.320 | -0.377 | -0.263 | 0.000 |  |  |  |  |  |
|  | G-4 - G-2 | -0.462 | -0.542 | -0.382 | 0.000 |  |  |  |  |  |
|  | G-4 - G-3 | -0.142 | -0.214 | -0.071 | 0.000 |  |  |  |  |  |

**Supplementary Table 6.** Morphological traits of α-genotypes in the *Vandenboschia radicans* complex in Korea and Japan (Ebihara et al. 2005).

|  | Korea | Japan | |
| --- | --- | --- | --- |
|  | *V. kalamocarpa* | *V. kalamocarpa* | *V. subclathrata* |
| Nuclear *GapCp* type | AA(A) | AA(A) | AA |
| Chloroplast *rbcL* type | Ⅰ, Ⅰ’’ | Ⅰ, Ⅰ’ | Ⅰ |
| Genomic formula | αα(α) | αα(α) | αα |
| Frond length | 3.1–3.3 cm | 3–13 cm | 2–5 cm |
| Frond width | 1.5–1.8 cm | 1.5–2.5 cm | 1.2–1.5 cm |
| Lamina | Almost flat or slightly waved wing | Wing crisped | Almost flat |
| Diameter of rhizome | 0.4 mm | 0.3–0.8 mm | ca. 0.3 mm |
